# The *cvn8* Conservon System Is a Global Regulator of Specialized Metabolism in Streptomyces coelicolor during Interspecies Interactions

**DOI:** 10.1128/mSystems.00281-21

**Published:** 2021-10-12

**Authors:** Bailey Bonet, Yein Ra, Luis M. Cantu Morin, Javier Soto Bustos, Jonathan Livny, Matthew F. Traxler

**Affiliations:** a Department of Plant and Microbial Biology, University of California, Berkeley, Berkeley, California, USA; b Infectious Disease and Microbiome Program, The Broad Institute of MIT, Cambridge, Massachusetts, USA; Georgia Institute of Technology

**Keywords:** *Streptomyces*, bacterial interactions, conservon, gene regulation, interspecies interactions, natural products, specialized metabolism

## Abstract

Interspecies interactions are known to activate specialized metabolism in diverse actinomycetes. However, how interspecies cues are sensed and ultimately lead to induction of specialized metabolite biosynthetic gene clusters remains largely unexplored. Using transcriptome sequencing (RNA-seq), we analyzed genes that were transcriptionally induced in the model actinomycete Streptomyces coelicolor during interactions with four different actinomycetes, including genes that encode unusual regulatory systems known as conservons. Deletions in one such system, encoded by the *cvn8* genes, led to altered patterns of pigmented antibiotic production by S. coelicolor during interactions. Further transcriptomic analysis of mutants lacking each of the five genes in the *cvn8* locus demonstrated that this system is a global regulator of at least four different specialized metabolite biosynthetic pathways. How conservon systems work at the mechanistic level to regulate gene expression is not well understood, although it has been hypothesized that they may function in a way similar to eukaryotic G-protein-coupled receptors. The data presented here indicate that the gene products of the *cvnA8* and *cvnF8* (SCO6939) genes likely function together in one part of the Cvn8 signaling cascade, while the *cvnC8* and *cvnD8* gene products likely function together in another part. Importantly, because *cvnD8* likely encodes a Ras-like GTPase, these results connect G-protein-mediated signaling to gene regulation in a bacterium. Additionally, deletion of any of the *cvn8* genes led to abnormally high expression of an adjacent cryptic lanthipeptide biosynthetic gene cluster, indicating that conservon systems may be fruitful targets for manipulation to activate silent specialized metabolite biosynthetic pathways.

**IMPORTANCE** Interactions between different species of actinomycete bacteria often trigger one of the strains to produce specialized metabolites, such as antibiotics. However, how this induction occurs at the genetic level is poorly understood. Using transcriptomic methods, we show that an unusual regulatory system, known as a conservon system, is responsible for regulating expression of multiple specialized metabolite biosynthetic gene clusters in the organism Streptomyces coelicolor during interactions. Conservon systems are unusual because they appear to employ small GTPases as an important component of their signaling cascades. Small GTPases are common in eukaryotic signaling pathways, but the results presented here are notable since they implicate a system that includes a small GTPase in global gene regulation in a bacterium. Mutants lacking this conservon system also showed abnormally high expression of a gene cluster involved in making an unknown specialized metabolite, suggesting that conservon mutants might be useful for driving natural product discovery.

## INTRODUCTION

Actinomycete bacteria produce a prodigious array of natural products (also known as specialized metabolites) from which many clinically important therapeutics have been derived. However, while these metabolites have been well studied for their applications in medicine, comparatively little is known about how they function in natural settings (1). Proposed roles for these metabolites range from interspecies signaling molecules to agents of interference competition.

There are many reported examples of microbial interspecies interactions that stimulate biosynthesis of specialized metabolites in actinomycetes. In one well-characterized example, the interaction of the fungus Saccharomyces cerevisiae with Streptomyces venezuelae resulted in production of the volatile compound trimethylamine (TMA) by the former, inducing an exploratory mode of growth in the latter (2). Jones and collaborators went on to show that TMA lowers the pH of the growth media, resulting in upregulation of iron transporters and secretion of a suite of siderophores by S. venezuelae (3). Though significant progress has been made toward understanding this interaction, the molecular details have yet to be elucidated. In another example, a recent study by Lee and coworkers demonstrated that a coculture of Myxococcus xanthus and Streptomyces coelicolor triggered the production of the antibiotic actinorhodin in the latter species as a result of iron competition (4). In yet another example, an interaction between *Amycolatopsis* sp. strain AA4 and S. coelicolor stimulated production of a novel antibiotic, amycomycin, by *Amycolatopsis* sp. AA4 (5). Additionally, results of multiple studies indicate that interspecies induction of antibiotic production is frequent and widespread (6–8). However, while this growing body of work illustrates that natural product biosynthesis is often connected to social interactions (6), the molecular and regulatory events that drive specialized metabolism during these interactions remain largely undefined. Identifying the relevant regulatory systems during interactions is of high interest given that they may represent targets for manipulation in future natural product discovery efforts.

The conservons are an enigmatic family of operons found mostly in actinobacteria. Conservons were first identified in the genome of S. coelicolor (9), where 13 variants of these conserved operons were found, prompting their designation as “conservons.” Typical streptomycetes usually have eight or more, while most mycobacteria have at least one conservon locus (10, 11). Each conservon consists of four genes encoding a predicted membrane protein with a histidine kinase-like ATPase domain (*cvnA*), a protein with a roadblock/LC7 domain typically involved in NTPase regulation (*cvnB*), a protein with a helix-turn-helix motif/conserved domain of unknown function (DUF 742) (*cvnC*), and a Ras-like GTPase (*cvnD*).

The first functional analysis of a conservon demonstrated that a fragment of a *cvnA* gene (renamed *rarA*) was sufficient to restore the formation of aerial hyphae in a mutant of Streptomyces griseus lacking a key response regulator (10). As noted above, CvnA proteins have regions of homology with histidine kinases, and the authors concluded that the truncated version of RarA was a constitutively active version of a sensor protein. A follow-up study of S. coelicolor subsequently showed that CvnA9 indeed possessed ATP hydrolysis activity and that a mutant lacking *cvnA9* exhibited qualitatively higher expression of three developmental genes (12). Importantly, Komatsu et al. (12) also showed that purified CvnD9 protein possessed GTPase activity, consistent with the notion that CvnD proteins may function as small GTPases in a signaling cascade. Finally, using bacterial two-hybrid assays, these authors also showed that CvnA9, -B9, -C9, and -D9 interacted with one another (12). These results, among others, led to the hypothesis that the conservons encode signaling systems similar to eukaryotic G-protein-coupled receptors (12). Additionally, these studies and more by Takano et al. observed that disruptions in some *cvn* loci (e.g., *cvn*s 1, 9, and 10 in S. coelicolor), especially mutation of *cvnA* genes, resulted in pleiotropic effects on specialized metabolism, with pigmented antibiotic production enhanced or diminished depending on the growth conditions (13). Thus, while at least some conservons appear to impact specialized metabolism, the extent of gene regulation controlled by conservons has not been explored, and their role in regulating specialized metabolism remains poorly defined.

In this report, we examine interspecies interactions that stimulate production of two pigmented antibiotics, actinorhodin and undecylprodigiosin, in S. coelicolor. A previous study demonstrated that interspecies interactions with other actinomycetes could stimulate the precocious synthesis of both of these molecules by S. coelicolor, along with a wealth of other specialized metabolites (14). Here we sought to investigate the S. coelicolor response to these interactions at the molecular level. Specifically, we aimed to identify regulatory systems involved in sensing interspecies interactions and to understand their role in regulating specialized metabolism, including production of pigmented antibiotics.

Using a series of transcriptomic analyses coupled with targeted mutations and pigmentation distribution analysis, we demonstrate that normal interspecies induction of pigmented antibiotics, specifically actinorhodin, requires the conservon 8 (*cvn8*) locus in S. coelicolor. Using transcriptome sequencing (RNA-seq), we found that *cvn8* influences the expression of at least four biosynthetic gene clusters, and thus, its gene products function as a global regulator of specialized metabolism during interactions. On the basis of global expression patterns, we conclude that the protein pairs of CvnA8 and CvnF8 (newly identified here), and CvnC8 and CvnD8 likely function together in different parts of the Cvn8 signaling pathway and that there is likely cross talk between the different Cvn8 proteins and other regulatory systems. Taken together, these results offer strong evidence that bacteria, in addition to eukaryotes, use G-protein-mediated signaling to control gene expression.

## RESULTS

### Conservon loci are upregulated during interspecies interactions.

**FIG 1:**
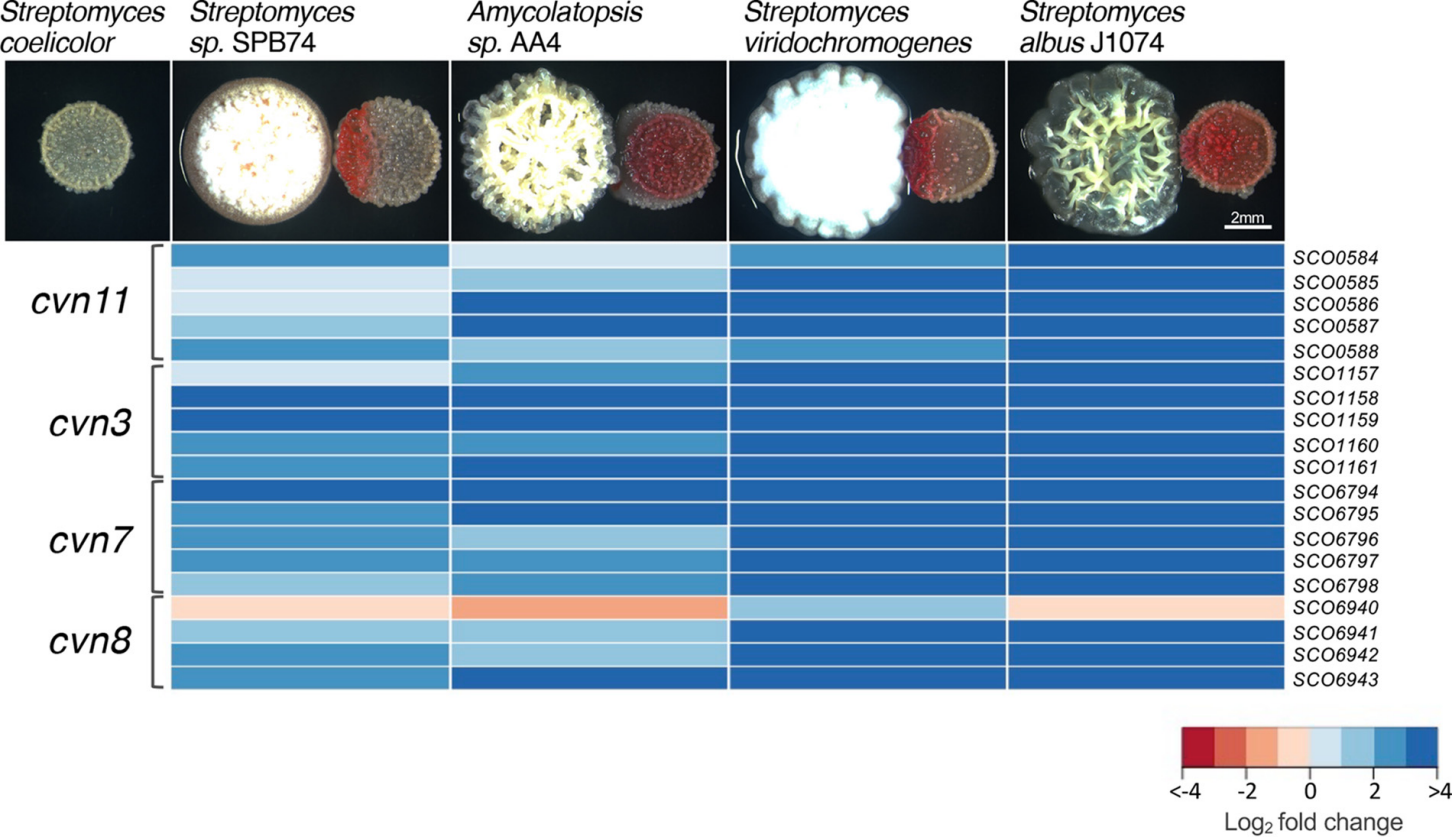
Conservon loci are upregulated in Streptomyces coelicolor during interspecies interactions. Phenotypes of S. coelicolor during growth as an isolated patch (top left) and during growth in interspecies interactions (right patch). Below each interaction image is a heatmap of the log_2_ expression ratio of four conservon loci in S. coelicolor during that interspecies interaction compared to S. coelicolor grown as an isolated patch.

Bacterial interactions have been previously shown to stimulate diversification of the S. coelicolor secreted metabolome (14). To gain a global view of the transcriptional response of S. coelicolor during interactions, we undertook an RNA-seq experiment. To set up the interactions, patches of S. coelicolor spores were plated 0.5 cm away from mycelial patches of the interacting strains on ISP2 agar plates and grown for 4 days before the halves of the S. coelicolor patches facing the interacting strains were harvested and their RNA was extracted. We also extracted RNA from S. coelicolor patches grown in isolation as a control. We chose to use *Streptomyces* sp. strain SPB74, *Amycolatopsis* sp. strain AA4, Streptomyces viridochromogenes, and Streptomyces albus J1074 as interacting strains because the secreted metabolome has been explored in these interactions (14). Additionally, these strains stimulated a range of production of red pigmented metabolites in S. coelicolor (Fig. 1). We note that at this pH, both actinorhodin and prodiginines appear as red pigmentation.

The expression profiles of the S. coelicolor patches grown in interactions were then compared to the expression profiles of the S. coelicolor patches grown in isolation to identify genes that might be involved in interactions, including the production of a diversified set of metabolites. We performed a Venn analysis to display genes that were differentially expressed in multiple interactions, since this might highlight genes that were generally involved in sensing and responding to interactions (see Fig. S1 in the supplemental material).

10.1128/mSystems.00281-21.1 FIG S1 Genes differentially expressed in S. coelicolor during interspecies interactions. Four-way Venn diagram showing S. coelicolor genes significantly upregulated in the interactions (A), the predicted localization or function of the genes significantly upregulated in all four interactions, determined by looking at their predicted domains or signal sequences (B). SM stands for specialized metabolite. Download FIG S1, PDF file, 0.3 MB. Copyright © 2021 Bonet et al. 2021 Bonet et al. https://creativecommons.org/licenses/by/4.0/ This content is distributed under the terms of the Creative Commons Attribution 4.0 International license.

When we investigated the genes that are differentially expressed in all four interactions, we found that there were 39 genes shared between all four interactions and 93 genes shared between three or more interactions (Fig. S1). For this data set, we considered genes to be differentially expressed if they had a log_2_ fold change greater or less than 2 standard deviations away from the average log_2_ fold change of the entire set of genes and had a *P* value of <0.05. Within the 39 genes that were differentially expressed in all four interactions, there were many genes encoding natural product biosynthesis machinery, predicted secreted or membrane-bound proteins, and several possible DNA binding proteins (Fig. S1). Many of the genes that we found to be most highly differentially expressed across the interactions were part of conservon loci, with the most positively expressed conservon gene being more than 7.7 log_2_ fold (or 200-fold) upregulated during the interaction (Fig. 1). Specifically, the responsive conservon loci included *cvn*s 3, 7, 8 and 11. These results indicated that these conservons might be involved in coordinating S. coelicolor’s response to interactions.

In addition to the conservon loci, several other regulatory genes were also significantly induced during these interactions. For example, all four interactions led to the induction of *scbA* and *rsfA*. ScbA is the primary gamma-butyrolactone synthase in S. coelicolor and is responsible for driving quorum sensing in this organism (15). Its induction here may indicate that all four interactions triggered enhanced production of quorum sensing signaling molecules. RsfA is an anti-sigma factor that is known to target the sporulation sigma factor, σ^F^ (16). Importantly, both ScbA and RsfA have been directly linked to regulation of pigmented antibiotics in S. coelicolor and thus will be of high interest for future study in these interaction systems.

### Conservon 8 is necessary for the timely induction of pigmented metabolites during interactions.

**FIG 2:**
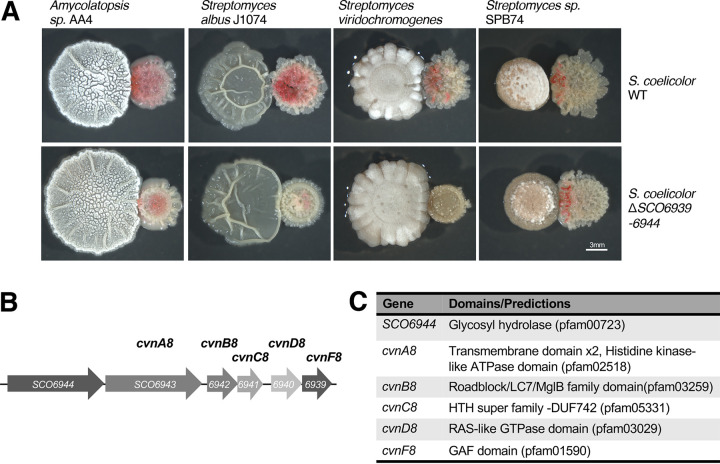
Conservon 8 is involved in the induction of pigmented metabolites during interactions. (A) Interspecies interactions of wild-type (WT) and Δ*cvn8* (Δ*SCO6939-6944*) S. coelicolor patches when grown in interspecies interactions with four interacting strains. Micrographs were taken after 3 days of growth with *Amycolatopsis* sp. AA4, after 4 days of growth with S. albus J1074 and after 5 days of growth with S. viridochromogenes and *Streptomyces* sp. SPB74. (B and C) The conservon 8 genomic locus (B) and the predicted domains within each gene (C) are shown.

In order to investigate the conservons further, we made targeted deletions of the entire conservon loci starting with *cvn7*, which led to no obvious change in the phenotype during interactions (Fig. S2). However, when we made a deletion of *cvn8*, we saw a strong delay and reduction in pigmentation during three of the four interspecies interactions (Fig. 2A), indicating that this conservon was involved in the timely induction of pigmentation during interspecies interactions. We note that for this experiment, the genes immediately upstream and downstream of the *cvn* genes were also deleted, since they appeared to be part of the same operon (Fig. 2B). *cvn8* has the four canonical genes *cvnA*, -*B*, -*C*, and -*D* that are found within all the conservons (Fig. 2C). We further characterized conservon 8 by analyzing the genomic structure and predicted protein domains for each gene. We note that *cvn3* and *cvn11* were not explored further.

10.1128/mSystems.00281-21.2 FIG S2 Δ*cvn7* interaction phenotypes. Interspecies interactions of wild-type (WT) and Δ*cvn7* (Δ*SCO6794-6797*) S. coelicolor patches when grown in interspecies interactions with four interacting strains. Micrographs were taken after 5 days of growth for every interaction shown. Download FIG S2, PDF file, 0.9 MB. Copyright © 2021 Bonet et al. 2021 Bonet et al. https://creativecommons.org/licenses/by/4.0/ This content is distributed under the terms of the Creative Commons Attribution 4.0 International license.

We named the gene immediately downstream of the core *cvn8* genes *cvnF* because our gene expression data indicated that it was coregulated with the *cvn8* genes. The *cvnF* gene encodes a protein with a single predicted GAF domain. GAF domains are typically involved in sensing small molecules, with the most well-studied GAF domains found in cyclic nucleotide phosphodiesterases (17). Directly upstream of the *cvnA* gene is a predicted glycosyl hydrolase, which we have yet to investigate further.

The CvnA8 protein is the second shortest CvnA protein out of the 13 total CvnA proteins encoded in the S. coelicolor genome. It has two predicted transmembrane helices (between residues 33 and 80) with eight predicted amino acids between them, likely indicating that CvnA8 lacks an extracellular sensor domain. Conservons 1 to 6 have CvnA proteins with extracellular nitrate/nitrite-type sensing domains located between the transmembrane domains. Additionally, initial analysis done by Komatsu et al. demonstrated that CvnA9 fractionated with the membrane fractions in their cell preparations. The CvnA8 protein has a predicted histidine kinase-like ATPase domain (pfam02518) which includes the key residues present in the catalytic and ATP-binding (CA) domain, indicating that it is likely a functional ATPase. Previous studies have shown that the CvnA9 protein has ATPase activity (12).

The CvnB8 protein has a predicted roadblock/LC7 domain which is known to modulate different functions of NTPases (18). Interestingly, the predicted structure of the CvnB8 protein is very similar to the Myxococcus xanthus MglB protein. The MglB protein is a well-characterized GTPase-activating protein (GAP) and has recently been found to have guanine nucleotide exchange factor (GEF) activity as well (19). This suggests that CvnB proteins likely play a role in regulating their cognate small GTPases (i.e., proteins encoded by the *cvnD* genes).

Like all CvnD proteins, CvnD8 is predicted to be a Ras-like GTPase. Ras proteins are small GTPases, typically found in eukaryotic systems, that participate in signal relay and regulation of critical cellular processes (20). The most well-studied bacterial Ras-like GTPase is the MglA protein in M. xanthus with which the CvnD8 protein shares 22% pairwise identity. The predicted catalytic and GTP binding domain of the CvnD8 protein has the canonical p-loop, switch I, and switch II regions along with other important residues for proper function, indicating that the CvnD8 protein is likely a functional GTPase.

CvnC proteins, including CvnC8, contain a domain of unknown function (DUF742) that is part of the helix-turn-helix (HTH) superfamily, suggesting that they may interact with DNA and possibly act as transcriptional regulators, thought this remains to be experimentally validated.

### The genes in conservon 8 contribute differentially to the distribution of pigment production across interacting S. coelicolor patches.

**FIG 3:**
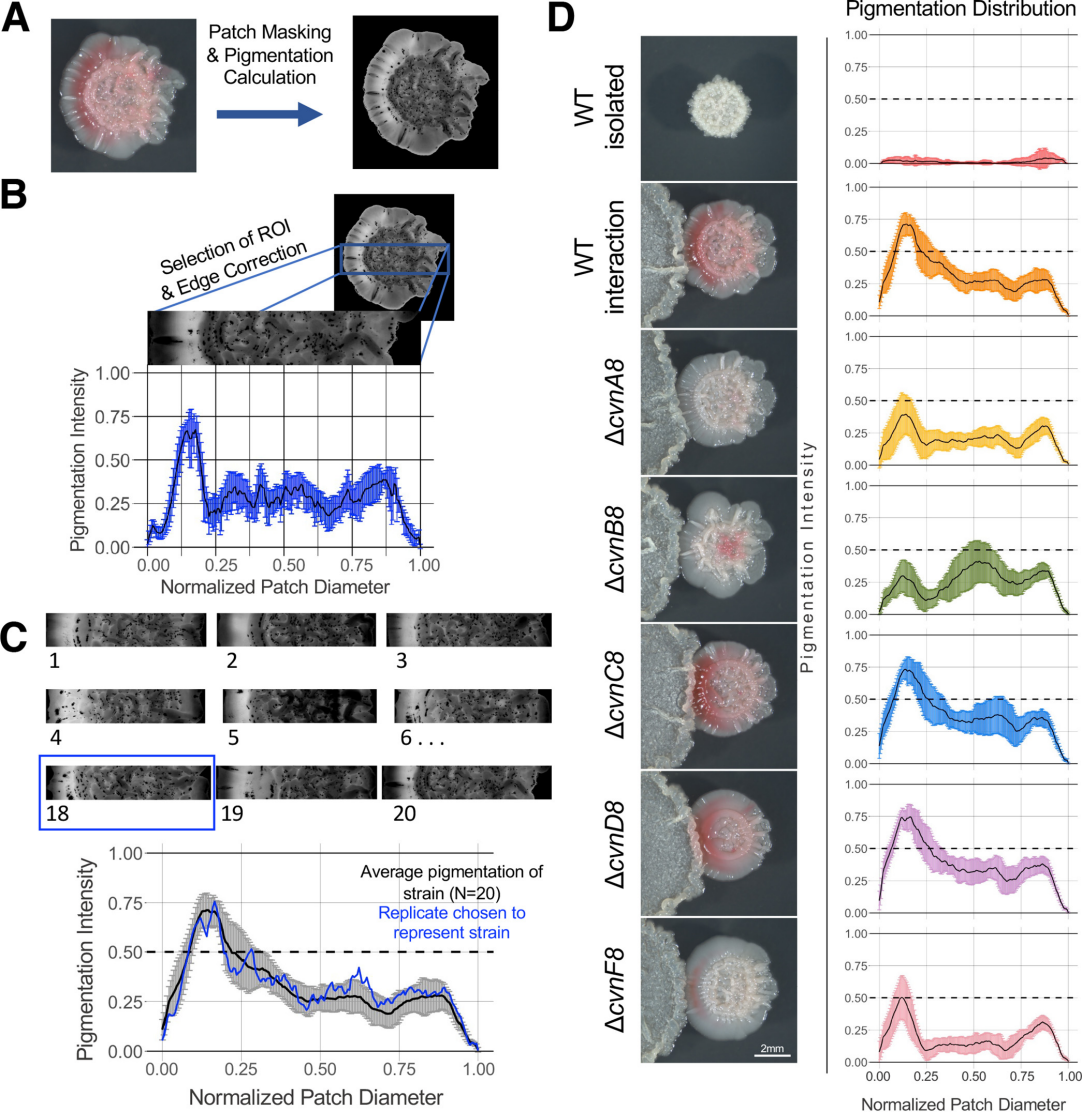
The conservon 8 genes contribute distinctly to the distribution of pigmentation across interacting S. coelicolor patches. (A) The input (left) and output (right) image from the patch masking and pigmentation detection method. (B) Within the rectangular region of interest (ROI), the pixels that fall into each breakpoint are averaged, normalized, and graphed along with the standard deviations (in blue). This calculation was done for 20 replicates of each S. coelicolor strain in an interaction with *Amycolatopsis* sp. AA4 and for 12 replicates of the wild-type S. coelicolor strain grown in isolation. (C) One representative replicate was selected based on its similarity to the average pigmentation distribution across all 20 replicates. (D) The representative replicates for each S. coelicolor strain are shown in the micrographs along with the pigmentation profile across the width of that patch.

After determining that *cvn8* was necessary for normal pigmented antibiotic production in S. coelicolor during interspecies interactions, we sought to examine the role that each individual conservon gene plays in this process. We constructed single gene deletions as described by Gust et al. (21). We hypothesized that doing so might result in a range of phenotypes in the single deletion strains. We placed the S. coelicolor single gene deletion strains in interactions with *Amycolatopsis* sp. AA4, spotting the spore patches 0.75 cm apart and growing them for 4 days at 30°C before observing the phenotype (Fig. 3D).

We developed a software package that enables quantification of the pigmentation intensity and distribution across S. coelicolor patches in a robust and unbiased manner (detailed in Materials and Methods and outlined in Fig. 3A to C). For each strain/condition, we averaged the pigmentation intensity across 20 replicate patches and chose the single replicate that best matched the average distribution as the representative image shown in Fig. 3D. We profiled the S. coelicolor wild-type strain as isolated patches and in interactions, along with all the single gene deletion mutants (Δ*cvnA8* to Δ*cvnF8*) in interactions. A pigmentation value of 0.5 or greater was consistent with easily visible pigmentation.

Wild-type S. coelicolor patches grown in isolation had almost no pigmentation. In contrast, the interacting wild-type strain displayed intense pigmentation in the first quarter of the patch, followed by a lighter pigmentation across the entire patch, decreasing in pigmentation at the right side of the patch. This is expected as pigmentation is induced in response to the interacting strain and the first quarter of the patch is closest to the interacting strain. We also observed a similar pattern in the Δ*cvnC8* and Δ*cvnD8* strains during interactions, with very robust pigmentation in the first quarter, followed by lighter pigmentation across the rest of the patch.

Interestingly, the Δ*cvnA8* and Δ*cvnF8* strains both had almost no pigmentation in the interactions, and they showed very similar distributions across the diameter of the patches (Fig. 3D). Δ*cvnF8* patches showed very slight pigmentation near the interaction edge of the patch, which can be seen in the pigmentation graph approaching the 0.5 intensity threshold. Δ*cvnB8* patches showed a unique pigmentation pattern, where the most intense pigmentation was located in the third quarter of the patch, in stark contrast to the wild-type S. coelicolor patches. Taken together, these differences in pigmentation distributions indicate that the individual genes of the *cvn8* locus contribute differentially to pigmentation production, with some mutations (e.g., *cvnA8*/*cvnF8* and *cvnC8*/*cvnD8*) leading to similar phenotypes.

### Expression patterns indicate that some subsets of Cvn8 proteins function together.

We next sought to understand how each of the conservon 8 proteins contributes to gene expression in S. coelicolor. To do so, we conducted RNA-seq analyses on the single gene deletion strains. We placed the S. coelicolor strains into interactions with *Amycolatopsis* sp. AA4, spotting the spore aliquots 0.75 cm apart on ISP2 agar and growing them for 4 days before harvesting the interaction-facing half of the interacting patch for RNA isolation. We also grew each mutant strain, and the wild type, as patches in isolation. We then compared the transcriptome of each mutant strain to the transcriptome of the wild-type strain grown under the same conditions (i.e., the wild-type transcriptome of isolated patches served as the control for the mutants grown in isolation, while the wild-type transcriptome obtained during interactions served as a control for comparison of the interacting mutant patches).

**FIG 4:**
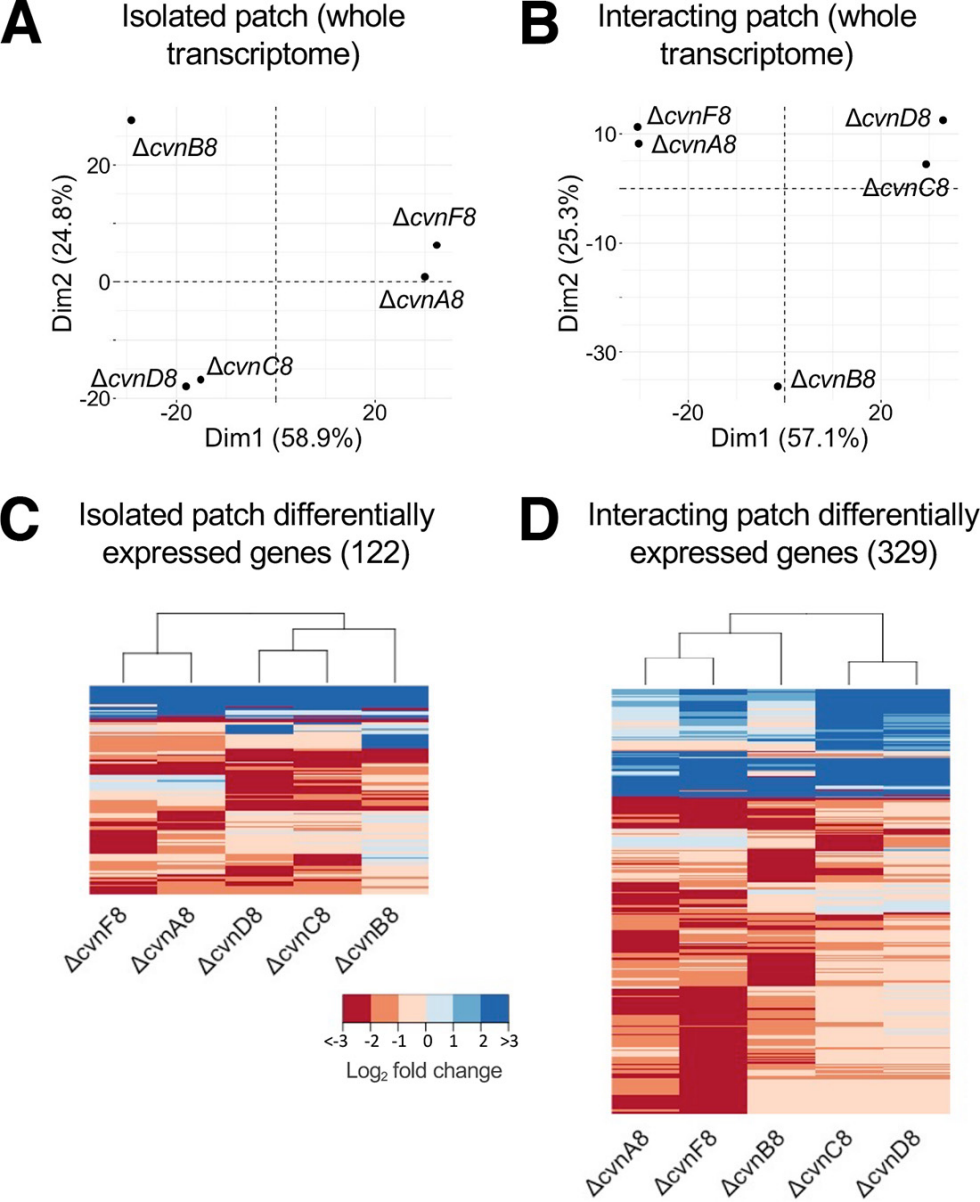
General expression patterns in conservon 8 mutants. (A and B) Using the log_2_ fold change of all the genes in the transcriptome, the principal components of each strain were calculated and plotted for the strains in isolated patches (A) and in interactions (B). Dim1, dimension 1. (C and D) Genes in these heatmaps were included if they showed an absolute log_2_ fold change of  ≥2 and a *P* < 0.05. The gene had to meet these criteria in at least one of the mutant strains to be shown. The dissimilarity of the samples was calculated and used to hierarchically cluster the strains shown in the dendrogram; 122 genes and 329 genes met these expression criteria in the isolated patches and the interacting patches, respectively.

To broadly visualize variation between the mutant transcriptomes, we conducted a principal coordinate analysis (PCA) on the average log_2_ fold change expression values for each mutant strain compared to the wild type, for both isolated patches (Fig. 4A) and for interacting patches (Fig. 4B). The first two principal components, or dimensions (“Dim”), explained a substantial proportion of the variation for both the isolated (83.7%) and interacting patches (82.4%), indicating that these PCAs capture the majority of the transcriptional changes within the data sets. Across both data sets, clear clustering patterns were observed. Namely, expression profiles for the Δ*cvnA8* and Δ*cvnF8* strains were tightly associated, as were the profiles for the Δ*cvnC8* and Δ*cvnD8* strains. In both PCAs, the profiles for the Δ*cvnB8* patches were distant from the profiles for the other strains, indicating that the transcriptional patterns associated with mutation of *cvnB8* were unique.

To obtain an idea of the global impact of deleting individual *cvn8* genes on the transcriptome, we examined genes that showed strong expression changes, i.e., at least 2 log_2_ fold (4-fold) change (either up or down) and a *P* value of <0.05 compared to wild-type (WT) expression under the same experimental conditions. Across all mutant strains, there were 122 genes that met these criteria when grown as isolated patches and 329 genes that met these criteria during interactions with *Amycolatopsis* sp. AA4, as shown in the heatmaps in Fig. 4C and D. The expression changes and functional annotation for these 329 genes are shown in Table S1 in the supplemental material. Across these 329 genes, after “hypothetical” with 105 genes, the most highly represented functional category was specialized metabolism, with 91 genes. Other notable functional categories included transport (34 genes) and regulation (18 genes). In Fig. 4C and D, the transcription profiles were hierarchically clustered by Euclidean distance, and once again, the Δ*cvnA8* and Δ*cvnF8* profiles grouped together, as did the profiles for the Δ*cvnC8* and Δ*cvnD8* strains. The profiles for the Δ*cvnB8* patches occupied an intermediate position in both hierarchical dendrograms, once again indicating that *cvnB8* mutants exhibited expression patterns that differed compared to the other two groups of mutants.

10.1128/mSystems.00281-21.8 TABLE S1 Annotations, functional categories, and fold change for genes shown in Fig. 4D. In the first tab (Genes_by_category), gene annotations are shown in column B. Functional categories are noted in columns C to I, with a Y indicating that a gene was counted in that category. The second tab (Gene_expression) contains log_2_ fold change data and *P* values for all genes in each mutant during interactions. Download Table S1, XLSX file, 0.1 MB. Copyright © 2021 Bonet et al. 2021 Bonet et al. https://creativecommons.org/licenses/by/4.0/ This content is distributed under the terms of the Creative Commons Attribution 4.0 International license.

Taken together, these broad analyses of the transcriptional profiles reinforce the patterns observed in our pigmentation distribution analysis. Namely, they suggest that CvnA8 and CvnF8 may have a similar, or tightly associated, function within the Cvn8 signaling cascade. Likewise, we hypothesize that CvnC8 and CvnD8 also likely function together at a similar step, with CvnB functioning in a different point within the Cvn8 regulatory cascade.

### The Cvn8 system controls the expression of pigmented antibiotic biosynthetic gene clusters.

**FIG 5:**
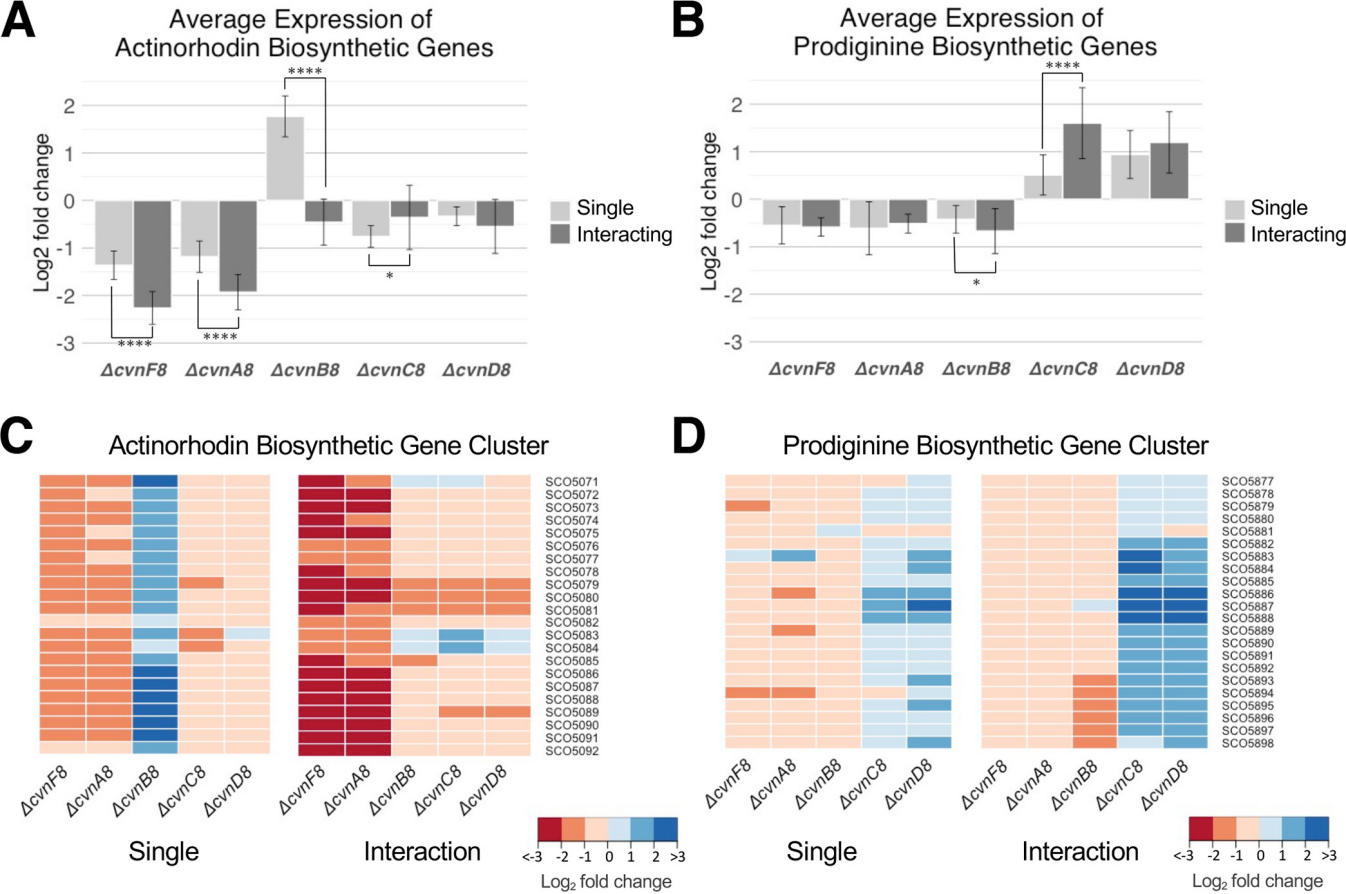
Influence of conservon 8 genes on the expression of pigmented specialized metabolite biosynthetic genes. (A and B) The average log_2_ fold change of the actinorhodin (A) and prodiginine (B) biosynthetic genes from transcriptomic data collected during growth as an isolated patch or in interactions. Error bars represent the standard deviation. Two-sample *t* tests were done, and significant differences between the isolated strain and interacting strain expression are shown (*, *P* ≤ 0.05; ****, *P* ≤ 0.0001). (C and D) Heatmaps of the individual genes in the biosynthetic gene clusters are shown with cool colors indicating upregulation and warm colors indicating downregulation.

We next sought to examine the impact of deleting individual *cvn8* genes on expression of genes involved in specialized metabolism. We considered genes significantly differentially expressed if they showed at least 1.58 log_2_ fold (3-fold) change (either up or down) and a *P* value of <0.05. Since deletion of the *cvn8* genes caused S. coelicolor to produce a reduced amount, and/or altered distribution, of pigmented specialized metabolites (actinorhodin and undecyl-prodigiosin), we first examined expression of these biosynthetic pathways across the different mutants. In order to gain insight on the broad changes across the gene clusters, we averaged the log_2_ fold expression values of all the genes within each pigmented antibiotic biosynthesis cluster for each mutant compared to the wild-type control grown in similar conditions (Fig. 5A and B). We also examined the log_2_ fold change in expression of each gene within the gene cluster (Fig. 5C and D).

Expression of the actinorhodin (*act*) biosynthetic gene cluster was strongly affected by mutations in the *cvn8* locus. Specifically, *act* expression was reduced in the Δ*cvnA8* and Δ*cvnF8* mutants (Fig. 5A and C). This was especially pronounced during interactions, where they showed an average log_2_ fold change of −2.26 and −1.93 (−4.8 and −3.81 fold change), respectively, across the entire cluster. This response directly correlates with the decrease in pigmentation observed in these strains during interactions (Fig. 3). The Δ*cvnB8* mutant exhibited a unique gene expression pattern for the actinorhodin biosynthetic gene cluster. In this mutant, the *act* genes were upregulated in isolated patches by an average of ∼3.5-fold but were not significantly differentially expressed when grown in interactions (Fig. 5A). We note that when we sampled biomass for RNA extraction, we harvested the half of the patch facing the interacting *Amycolatopsis*, and thus, we did not sample the third quarter of the Δ*cvnB8* patch that showed abnormally high pigmentation. The Δ*cvnC8* and Δ*cvnD8* strains showed slight downregulation of the actinorhodin biosynthetic genes, which was not considered significant (log_2_ fold < 1 or > −1). Taken together, these data indicate that CvnB8 contributes to the negative regulation of *act* expression in isolated patches and that CvnA8 and CvnF8 are required for normal induction of *act* during interactions (see Fig. 7).

For the prodiginine (*red*) biosynthetic gene cluster, we observed only slightly lowered expression in the Δ*cvnA8*, Δ*cvnF8*, and Δ*cvnB8* strains irrespective of the growth conditions (Fig. 5B and D). In contrast, the Δ*cvnC8* and Δ*cvnD8* strains exhibited increased expression of the *red* gene cluster in both isolated and interacting patches. This abnormally high expression was particularly pronounced in the Δ*cvnC8* mutant, where the upregulation during the interaction was significantly higher than the upregulation in the isolated patches. The high expression of the *red* genes in the Δ*cvnC8* and Δ*cvnD8* strains reinforces the finding in the pigmentation analysis (Fig. 3) in which these strains showed robust pigmentation during the interactions. These data indicate that CvnC8 and CvnD8 contribute to negative regulation of the prodiginine biosynthesis genes, with CvnC having the stronger effect during interactions (see Fig. 7).

### The Cvn8 system controls the expression of additional specialized metabolite biosynthetic gene clusters.

**FIG 6:**
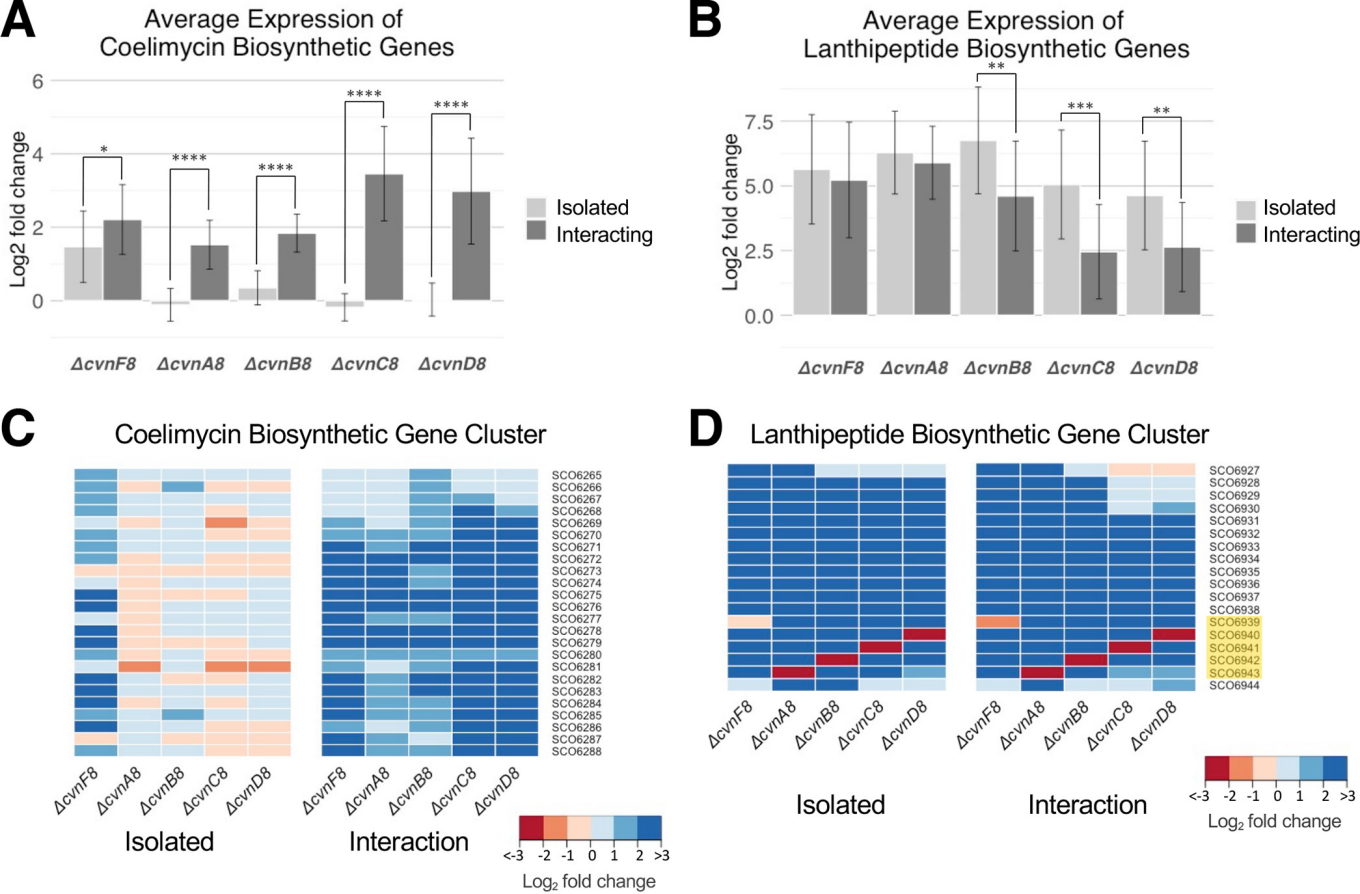
Influence of conservon 8 genes on the expression of additional specialized metabolite biosynthetic genes. (A and B) The average log_2_ fold change of the coelimycin (A) and an unknown lanthipeptide (B) biosynthetic genes from transcriptomic data collected during isolated patch growth and interaction growth. Error bars represent the standard deviations. Two-sample *t* tests were done, and significant differences between the isolated strain and interacting strain expression are shown (*, *P* ≤ 0.05; **, *P* ≤ 0.01; ***, *P* ≤ 0.001; ****, *P* ≤ 0.0001). (C and D) Heatmaps of the individual genes in the biosynthetic gene clusters are shown with cool colors indicating upregulation and warm colors indicating downregulation. Highlighted genes belong to the *cvn8* cluster (D).

In addition to the prodiginine and actinorhodin gene clusters, the *cvn8* mutants exhibited changes in the expression of two other specialized metabolite biosynthetic gene clusters. This altered gene expression included significant upregulation of the coelimycin biosynthetic gene cluster, *cpk*, in all five of the single gene deletion mutants during interactions (Fig. 6A and C). While the change in gene expression in the *cpk* cluster in each of the mutants was in the same direction, the magnitude of the change differed. The Δ*cvnC8* and Δ*cvnD8* strains exhibited a larger increase in average expression of ∼11-fold and ∼7.9-fold, respectively, compared to the lesser induction in the Δ*cvnF8*, Δ*cvnA8*, and Δ*cvnB8* strains (∼4.6-, ∼2.9-, and ∼3.6-fold, respectively). The only mutant that displayed any change to the expression of the coelimycin biosynthetic gene cluster in isolated patches was Δ*cvnF8*. Taken together, these results indicate that the Cvn8 system plays a role in negatively regulating the *cpk* gene cluster during interspecies interactions.

The most highly differentially expressed genes in our transcriptomic data sets are from a cryptic lanthipeptide biosynthetic gene cluster, which we term the *lan* cluster (SCO6927-SCO6938). No small molecule has been associated with the *lan* gene cluster, and the *cvn8* locus is directly adjacent to the *lan* genes, indicating that they may have an important direct connection.

**FIG 7:**
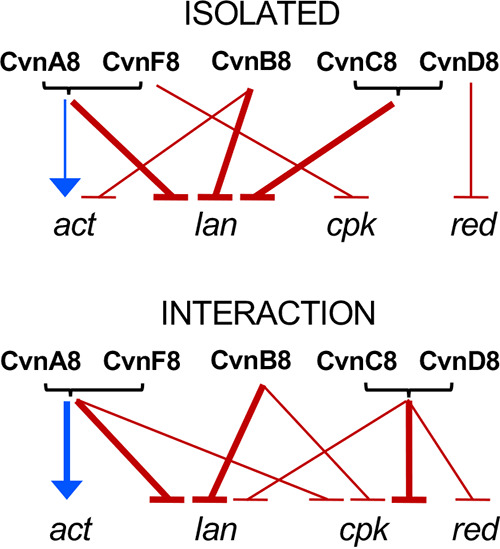
Summary of regulation of specialized metabolite biosynthesis genes. The effect of each conservon 8 gene on four specialized metabolite biosynthetic gene clusters determined from transcriptomic analysis: actinorhodin (*act*), prodiginine (*red*), coelimycin (*cpk*), and the cryptic lanthipeptide (*lan*). Blue arrows indicate positive regulation, and red arrows indicate negative regulation. Thicker arrows indicate >2 log_2_ fold change, and thinner arrows indicate between 1 and 2 log_2_ fold change.

The two *lan* genes encoding the precursor peptides, SCO6931 and SCO6932, were both strongly upregulated (∼600-fold) in the Δ*cvnB8* strain compared to the wild type during growth as an isolated patch, highlighting the acute upregulation we observed in the *lan* cluster. Furthermore, the *lan* genes were upregulated on average >37-fold in the Δ*cvnA8* and Δ*cvnF8* strains, irrespective of whether they were grown in isolation or in interactions. This implies that CvnA8 and CvnF8 contribute significantly to the negative regulation of the *lan* biosynthetic gene cluster regardless of the presence of an interacting strain (Fig. 7). Likewise, CvnB8, CvnC8, and CvnD8 also play a role in negatively regulating the *lan* genes in both the presence and absence of an interacting strain, but they appear to exert a stronger effect in the absence of the interaction (Fig. 6B). This indicates that the Cvn8 system strongly represses the expression of the *lan* genes without any interaction stimulus. Like the rest of the *lan* cluster, the adjacent *cvn8* genes (Fig. 6D, highlighted in yellow) themselves exhibited aberrantly strong expression in all the mutant strains, indicating that the *cvn8* genes are negatively autoregulated.

## DISCUSSION

The stimulation of specialized metabolism during interspecies interactions between actinomycete bacteria is a well-documented phenomenon. However, how the cues exchanged during these interactions lead to the activation of specialized metabolite biosynthetic gene clusters is poorly understood at the genetic level. In this work, we sought to identify and investigate the regulatory system(s) responsible for the interspecies activation of specialized metabolism in the model actinomycete Streptomyces coelicolor.

Using a transcriptomic approach, we found that multiple sets of regulatory operons, known as conservons, were induced during interspecies interactions between S. coelicolor and four other actinomycetes. Conservon systems share a conserved gene cohort that includes a small Ras-like GTPase. Deletion of the locus encoding one of these conservon systems, *cvn8*, led to a delay in the typical production of two pigmented antibiotics during interactions, indicating that *cvn8* is a key regulatory system in this context (Fig. 2A). We subsequently deleted each of the five genes of the *cvn8* locus. The pigmentation phenotypes and genome-wide transcriptome profiles of these mutants point to a model in which specific Cvn8 proteins function together at discrete steps to regulate specialized metabolism. Deletion of *cvn8* genes led to aberrant expression of the biosynthetic gene clusters for actinorhodin, prodiginine, coelimycin, and a cryptic lanthipeptide (Fig. 7). Thus, the Cvn8 system functions as a pleiotropic global regulator of specialized metabolism during interspecies interactions. Beyond this, these results connect G-protein-mediated signaling to global genetic regulation in a bacterium.

### A foundation for a revised model of conservon function.

Previous studies have established that conservon systems can affect both development and secondary metabolism in *Streptomyces* (10, 12, 13). The most in-depth biochemical study conducted to date by Komatsu et al., showed that the CvnA9 protein, which was hypothesized to be a membrane-bound sensor protein, had ATPase activity and the ability to interact with the CvnB9 and CvnC9 proteins (12). *cvnD9*, like all *cvnD* genes, is predicted to encode a Ras-like GTPase. Importantly, Komatsu et al. also demonstrated that purified CvnD9 had GTPase activity, and interacted with CvnB9 and CvnC9. On the basis of these results, Komatsu et al. hypothesized that the proteins of the Cvn9 system form a membrane-associated heterocomplex that parallels eukaryotic G-protein-coupled regulatory systems involved in outside-in signaling. In this model, CvnA9 serves as the sensor protein which is bound by CvnB9 and CvnC9. In turn, CvnB9 and CvnC9 also bind CvnD9-GDP. It was further hypothesized that activation of CvnA9 ultimately led to the exchange of GDP for GTP in the CvnD9 active site, and the release of CvnD9-GTP from the membrane complex which might activate downstream effector proteins to modulate gene expression. The results we present here, along with updated protein predictions, prompt a refinement of this model.

### Connected function of CvnA and CvnF.

The results presented in this work suggest the cooperation of CvnA8 and an additional protein encoded by the gene directly downstream of *cvnD8*, newly named here as *cvnF8* (SCO6939). One piece of evidence for this is the strong reduction in pigmentation exhibited in both the *cvnA8* and *cvnF8* deletion mutants when in interactions with *Amycolatopsis* sp. AA4 (Fig. 3D). A second piece of evidence is that the transcriptomes of the *cvnA8* and *cvnF8* deletion mutants largely paralleled one another at both the global level and when the four specialized metabolite biosynthetic gene clusters examined here are considered (Fig. 4 and 6 and summarized in Fig. 7).

Another possible clue that the CvnA8 and CvnF8 proteins might work together comes from analysis of the predicted structure of the CvnA8 protein, along with the genes found downstream of the *cvnD* genes. In S. coelicolor, the CvnA predicted proteins from conservons 1 to 6 have clear extracellular, nitrate/nitrite sensing (NIT)-type domains (pfam08376). In contrast, the CvnA7 to -13 proteins have very short regions (<20 residues) between the two transmembrane domains, indicating they do not contain extracellular sensor domains. Conservons 7 and 8 each have a gene encoding a GAF domain-containing protein (e.g., *cvnF8*) downstream of *cvnD*, while conservons 9 to 12 have genes encoding cytochromes p450 (previously termed *cvnE* genes). One common function of GAF domains is the binding of small molecules and the subsequent allosteric regulation of enzyme activity, often of regulatory domains with NTPase/NTP modification activity (17). This, combined with our data showing that the deletion of the *cvnA8* and *cvnF8* genes leads to tightly parallel sets of complex phenotypes, leads us to hypothesize that the CvnF8 protein may act as the sensory input for CvnA8. Further exploration of the sensory aspects of CvnA/F8 will be required to test this relationship.

### Connected function of CvnD and CvnC.

The findings presented here also suggest that CvnC8 and CvnD8 may function together to affect gene expression and pigmentation in S. coelicolor during interspecies interactions. In contrast to the Δ*cvnA8* and Δ*cvnF8* strains, both the Δ*cvnC8* and Δ*cvnD8* strains showed no reduction in pigmentation intensity in interactions with *Amycolatopsis* sp. AA4 (Fig. 3D). In fact, in these mutant patches during interactions, the prodiginine biosynthesis (*red*) gene cluster was robustly upregulated compared to the wild-type strain. Gene expression patterns in the *cvnC8* and *cvnD8* patches tightly matched each other, and this pattern was evident at the genome scale (Fig. 4) and at the level of individual specialized metabolite biosynthetic gene clusters (summarized in Fig. 7). On the basis of these results, we hypothesize that CvnC8 and CvnD8 likely act in concert within the Cvn8 regulatory cascade.

The primary structure of the CvnD8 protein possesses features of a functional Ras-like GTPase, with the typical predicted topology and almost all of the conserved residues important for guanine nucleotide binding. Small GTPases are common in eukaryotic signaling systems where they function as molecular switches in many archetypal regulatory cascades. A recent analysis showed that CvnD proteins belong to the MglA family of small Ras-like GTPases (11). In M. xanthus, MglA has been shown to function as a switch that regulates cell polarity and gliding motility (20, 22–24). In keeping with Komatsu et al., we presume that signaling through the Cvn8 system depends on the nucleotide (i.e., GTP or GDP) bound by CvnD8.

The *cvnC8* gene encodes a small, 136-residue protein with a conserved domain of unknown function (DUF742) in the helix-turn-helix (HTH) superfamily. Helix-turn-helix motifs are typically involved in binding nucleic acids, for example in transcriptional regulatory proteins. Due to the comprehensive similarity in the *cvnC8* and *cvnD8* mutant phenotypes and transcriptional patterns, we hypothesize that CvnD8 and CvnC8 act together in the same step in the regulatory cascade. One appealing hypothesis is that activated CvnD8-GTP might bind to CvnC to form a DNA binding complex, which then represses expression. This repression is particularly apparent in the regulation of the *lan* genes, which were strongly expressed in all the *cvn* mutants analyzed here.

### Potential function of CvnB.

Among the mutants investigated in this study, the Δ*cvnB8* strain showed the most unique set of phenotypes and gene expression patterns. The *cvnB8* mutant produced an intermediate amount of pigmented antibiotics, but this pigmentation was mislocalized, e.g., not near the interaction edge, but instead peaking in the third quarter of the interacting patch (Fig. 3D). When we sampled interacting patch biomass for RNA extraction, we harvested the half of the S. coelicolor patch facing the interacting *Amycolatopsis* patch. As a result, we did not analyze RNA from the areas of the Δ*cvnB8* patches exhibiting mislocalized pigmentation, and thus our ability to address the causes of this unusual pattern is limited. At a global level, the Δ*cvnB8* strain transcriptomes were also unique, as seen in the PCA analyses in Fig. 4. At the level of the four biosynthetic gene clusters examined here, the Δ*cvnB8* strain shared transcriptional patterns with the Δ*cvnA8* and Δ*cvnF8* strains for some gene clusters (e.g., the *red* genes; Fig. 5B), and with the Δ*cvnC8* and Δ*cvnD8* strains for others (e.g., the *lan* genes; Fig. 6B).

A new piece of information that has come to light since the last proposed conservon mechanistic model (12) is that CvnB proteins share homology with the MglB protein of Deltaproteobacteria (11). In M. xanthus, MglB has been shown to act as a GTPase-activating protein (GAP) for the Ras-like GTPase MglA (22). GAPs typically bind to the small GTPases they regulate and stabilize the active site to increase the rate of GTP hydrolysis, returning the small GTPase to the GDP-bound, inactive form. Intriguingly, the MglB protein was recently shown to also act as a guanine nucleotide exchange factor (GEF) (19). GEFs promote the exchange of GDP for GTP in the GTPases they regulate to switch them from their inactive form to their active form.

Given the extremely strong derepression of the *lan* gene cluster in the *cvn8* mutant strains, we hypothesize that this cluster is a direct target of this regulatory system. If CvnB8 acted primarily as a GAP to inactivate CvnD8, we would predict that the Δ*cvnB8* and Δ*cvnD8* strains would show opposing gene expression patterns of the *lan* genes. However, since both the Δ*cvnB8* and Δ*cvnD8* strains showed similar derepression of the *lan* genes, we hypothesize that CvnB8 primarily functions as a GEF that toggles CvnD8 to its active, GTP-bound form.

### Towards an updated model of Cvn function.

What updates to the model of Cvn function are prompted by the data presented here? The original model by Komatsu et al. (12) proposed that CvnA9 is membrane bound and that CvnB9, -C9, and D9-GDP associate with CvnA9. After receipt of the stimulus by CvnA, the signal is relayed through the CvnB9 and CvnC9 proteins to CvnD9, which is ultimately released in a GTP-bound (active) form to interact with downstream regulatory proteins, including, presumably, DNA binding proteins. On the basis of the output of DNA expression patterns observed for each Cvn8 mutant presented here, along with updated protein functional predictions, we considered whether or not elements of the Komatsu model could be revised for the Cvn8 system. In doing so, we limited our model to consideration of the effect of the Cvn8 system on the *lan* genes, since the magnitude of transcriptional changes detected in the mutants and the proximity of the *cvn8* genes to the *lan* genes both point to a direct role for the Cvn8 system in regulating this gene cluster.

**FIG 8:**
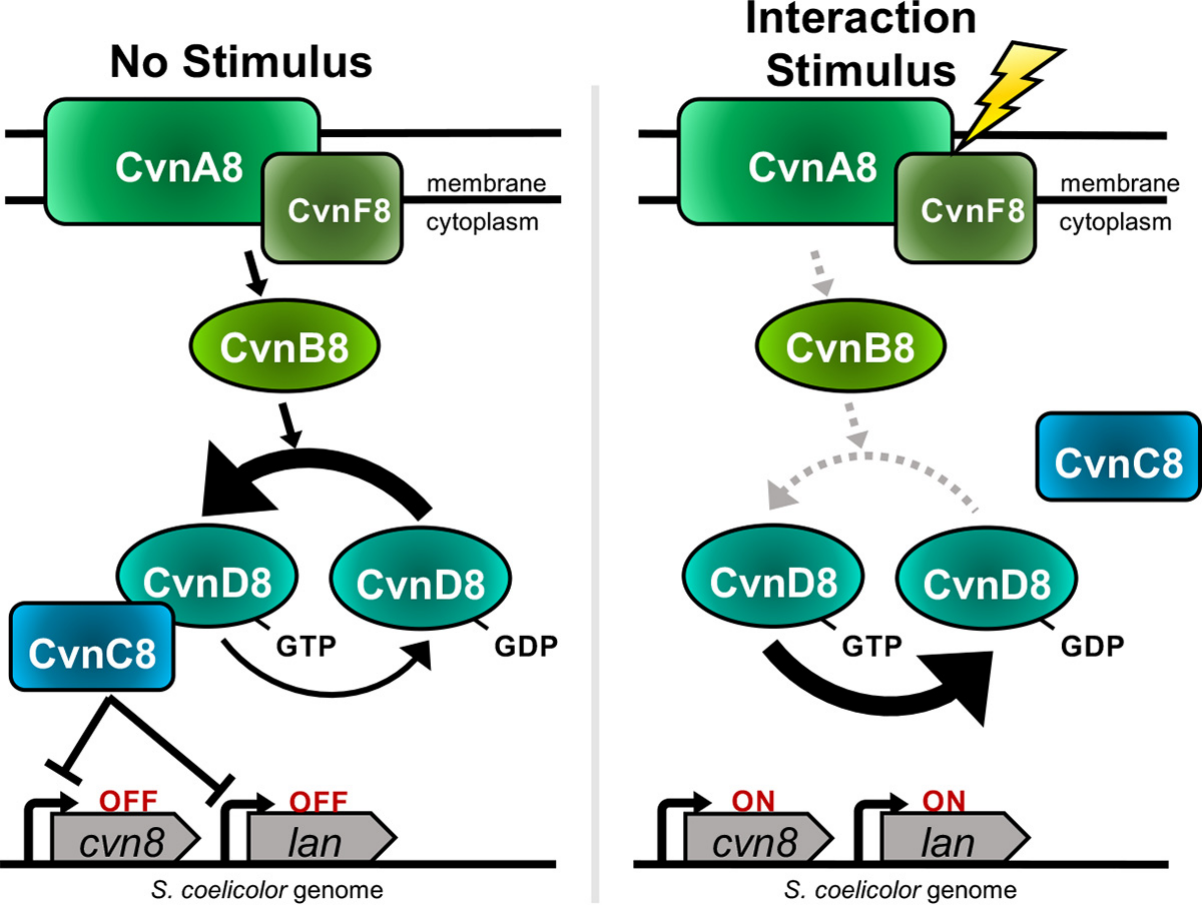
Hypothetical model of Cvn8 system function. Black arrows indicate active signal flow; gray dashed arrows represent inactive connections. The large arrow indicates the major direction of GTP/GDP switching of CvnD8 under each condition. In the presence of the interaction stimulus, *cvn8* and *lan* genes are ultimately derepressed as a result of CvnC8/CvnD8 dissociation. The positions of CvnA/F and CvnC/D relative to one another are arbitrary.

The aberrantly high expression of the *lan* genes seen in all the mutants examined here points to the primary role of the Cvn8 system as a repressor of expression of these genes. Thus, in the absence of a stimulus, the default state of the Cvn8 system is one in which a repressor, which we hypothesize includes CvnC8, is bound to DNA (Fig. 8). However, binding of the repressor is contingent on all parts of the Cvn8 system being present, since deleting any one of them results in derepression. To account for this activity, we propose that in the absence of any stimulus, CvnA8/F8 actively signals to the downstream Cvn proteins, likely CvnB8 (which we propose acts as a GEF in this scenario), to convert CvnD8-GDP to the active GTP-bound form. CvnD8-GTP might then work together with CvnC to repress *lan* expression. Removing any member of this signaling cascade would result in derepression of the *lan* genes, which is consistent with the mutant data presented here. Beyond this, since the *cvn8* genes are autorepressed, this default activity of the Cvn8 system would function to keep expression of the *cvn8* genes low in the absence of any stimulus.

This model also proposes that when the stimulus is present, e.g., during interspecies interactions, signaling via CvnA8/F8 and CvnB is interrupted. Without constant conversion to the GTP-bound form, CvnD8’s intrinsic GTPase activity would ensue, rendering it unable to interact with CvnC8 to effectively repress *lan* gene expression. While this model accounts for the expression changes observed in the *cvn* mutant strains and is consistent with current protein functional predictions, we present it here only as a hypothetical framework. We emphasize that a range of future studies will be required before a full understanding of Cvn8 system functionality is achieved.

### Potential cross talk with other systems.

With 13 conservons present throughout the S. coelicolor genome, it seems likely that there may be cross talk between these systems. Not all the transcriptomic regulation across the four biosynthetic gene clusters emphasized here can be explained by regulation via the Cvn8 system alone. For example, during interactions, the Δ*cvnA8* and Δ*cvnF8* strains were the only *cvn8* mutants that showed a significant change in expression of the *act* genes. This implies that the CvnA8 and CvnF8 proteins have an independent effect on the *act* genes that does not involve the other members of the Cvn8 system. We hypothesize that this effect is the result of regulation flowing through other proteins, possibly other CvnB, -D, and -C proteins, via molecular cross talk. Additionally, only the deletions of the *cvnC8* and *cvnD8* genes had an effect on the expression of the prodiginine (*red*) biosynthetic genes, which indicates that the effect of the CvnC8 and CvnD8 proteins on these genes is independent of the other Cvn8 proteins. In this case, we speculate that other CvnA and -B proteins may provide upstream inputs for CvnC8 and CvnD8. Across the 13 conservons in S. coelicolor, the CvnD protein is the most well conserved, and the CvnB8 protein is the second. In contrast, the CvnA8 protein shows the most variability as a result of the different signaling domain configurations discussed above. Thus, the possibility of cross talk and redundancy make the conservon systems challenging to dissect from a functional perspective. Identifying exclusive target genes that can serve as outputs for monitoring activity within specific Cvn systems is therefore a critical step toward designing future experiments that address Cvn functionality.

### Concluding remarks.

This work demonstrates that signaling via the Cvn8 system influences the expression of multiple specialized metabolite biosynthetic gene clusters in S. coelicolor. Moreover, this regulatory system plays a pleiotropic role in coordinating expression of these genes in the context of interspecies interactions. Given that the Cvn8 system likely involves G-protein-mediated signaling, this work strongly indicates that this signaling modality is used by eukaryotes and bacteria alike to regulate gene expression. Beyond this, the enhanced expression of a cryptic specialized metabolite biosynthetic gene cluster (*lan*) in mutants lacking *cvn8* genes suggests that conservon systems might be fruitful targets for future studies aimed at novel compound discovery.

## MATERIALS AND METHODS

### Bacterial strains and growth conditions.

All bacterial strains used in this study are listed in Table 1. Strains of Escherichia coli were grown at 37°C in LB medium with the appropriate antibiotics, if necessary. Strains of Streptomyces coelicolor were grown on ISP2 agar (10 g malt extract, 4 g glucose, 4 g yeast extract, 15 g agar, 1 liter double-distilled water [ddH_2_O]). *Streptomyces* spores were generated and harvested using standard methods (25).

**TABLE 1:** List of bacterial strains used in this study

Strain	Relevant genotype^*a*^	Use	Reference
E. coli ET12567/pUZ8002	*dam tra* genes	*Streptomyces* conjugation	MacNeil et al. (26)
E. coli/St1G8	S. coelicolor gDNA: 7674874−7712565 bp	gDNA cosmid with *cvn8*	Redenbach et al. (27)
E. coli BW25113/pIJ790	λ RED genes	Recombining the targeting cassette	Datsenko and Wanner (28)
E. coli DH5α/pIJ773	*aac(3)IV*, *oriT* (RK2), FRT sites	Apramycin cassette amplification	Gust et al. (21)
S. coelicolor M145 strains			
* * Wild-type	Wild-type, SCP1^−^ SCP2^−^		Kieser et al. (25)
* ΔcvnA8* mutant	*cvnA8*::*acc(3)IV*		This study
* ΔcvnB8* mutant	*cvnB8*::*acc(3)IV*		This study
* ΔcvnC8* mutant	*cvnC8*::*acc(3)IV*		This study
* ΔcvnD8* mutant	*cvnD8*::*acc(3)IV*		This study
* ΔcvnF8* mutant	*cvnF8*::*acc(3)IV*		This study
* Δcvn8* mutant	*cvn8*::*acc(3)IV*		This study

a gDNA, genomic DNA; FRT, FLP recombination target.

### Plating interactions.

S. coelicolor spore stocks were diluted to 2 × 10^8^ CFU/ml, and 0.5 μl was spotted onto a 60 × 15-mm petri dish with 4 ml ISP2 agar, resulting in agar approximately 2 mm thick. For interactions, 0.5 μl of an *Amycolatopsis* sp. AA4 stock (8 × 10^8^ CFU/ml) was spotted 0.75 cm away from the S. coelicolor spot. Interactions were grown for 4 days at 30°C and then imaged or harvested for RNA isolation.

### Generation of deletion mutants.

Deletion mutants were generated in S. coelicolor M145 using the ReDirect PCR targeting system and the cosmid St1G8 (21). Deletions were verified using PCR spanning the deletion followed by sequencing of the PCR product for confirmation. They were also confirmed with a PCR within the deleted gene, resulting in no PCR product. All primers used are listed in Table 2.

**TABLE 2:** List of primers used in this study

Gene	Primer^*a*^	Sequence^*b*^
Primers for generating the PCR targeting cassette		
** ***cvn8* targeting cassette	FW	CTCGCTTAGCCACCCACCCCCCTGACGAGGATTCCCATGATTCCGGGGATCCGTCGACC
	RV	GCGGCGTGCGGTAGGGGTCGCTGGGGTGCTGTGACATCATGTAGGCTGGAGCTGCTTC
** ***cvnA8* targeting cassette	FW	CTCGCTTAGCCACCCACCCCCCTGACGAGGATTCCCATGATTCCGGGGATCCGTCGACC
	RV	GCCAGTGGATGTGCCGGGCCACCGGGAACGGACGGGTCATGTAGGCTGGAGCTGCTTC
** ***cvnB8* targeting cassette	FW	CATCCACTGGCCCTTCCCCCTTCTGGAGCGATCCCTATGATTCCGGGGATCCGTCGACC
	RV	ACGCCGACTCCTCGGCCGCGCGGCCGTACGTGCTCACCGTGTAGGCTGGAGCTGCTTC
*cvnC8* targeting cassette	FW	CCCCGTCCGCGTTGGCACGGGCGGCGAGGTCCGGTGAGCATTCCGGGGATCCGTCGACC
	RV	GCACCGGCCCGCGGGTGCGGCGAAACCGTGGACGGGTCATGTAGGCTGGAGCTGCTTC
*cvnD8* targeting cassette	FW	CACCCGCGGGCCGGTGCGCCGACCGTGCTGAAGATCATGATTCCGGGGATCCGTCGACC
	RV	GCGGCGTGCGGTAGGGGTCGCTGGGGTGCTGTGACATCATGTAGGCTGGAGCTGCTTC
*cvnF8* targeting cassette	FW	TGGTCACACCCCTTCTACCACCCTCCAGGACGCCTGATGATTCCGGGGATCCGTCGACC
	RV	ATCGCCTACGGCTCCCGGGTGGGGGTGTGGAACGGCTTATGTAGGCTGGAGCTGCTTC
Primers to verify gene deletion		
* cvn8*	FW	CCGACGACCTGGCTAGTGCTTC
	RV	GGTGATCACCCACTCGTTGCC
* cvnA8*	FW	CACATGGGCCTGATCCAGAG
	RV	GTGGGTACTCATAGGGATCGC
	RV2	GGATCAGCGACATGATGGCC
* cvnB8*	FW	CACATCCACTGGCCCTTCCC
	RV	GAGACGAGAACTTTGACGGCG
	RV2	ACGGGGATCACCATGTACGG
* cvnC8*	FW	CCTGTTCTTCGTCCAGAGCG
	RV	ATGTCCTCGAAAAAGCTGACGG
	RV2	GAGACGAGAACTTTGACGGCG
* cvnD8*	FW	AAGAGGAAGTGGCCTGATGC
	RV	AGGTCGTCGAAGTCCTCGTT
	RV2	ATGTCCTCGAAAAAGCTGACGG
* cvnF8*	FW	CTTGGAGAGCAGCTTCACGCC

a FW, forward; RV, reverse; RV2, reverse primer 2.

b Underlined sequences correspond to DNA from the *S. coelicolor* genome in hybrid primers.

To examine whether the deletions were polar, we observed the expression of the genes surrounding the deletion cassette in our transcriptomic data. We found that the surrounding genes were expressed without issue. In fact, the immediately surrounding genes in all cases were expressed at higher levels in the mutant strains compared to the wild type (see Fig. S4 in the supplemental material). To further evaluate whether or not introduction of the *aac(3)IV* cassette influenced downstream gene expression as a result of readthrough from promoters within the *aac(3)IV* cassette, we examined the RNA-seq coverage data for signs of readthrough transcription (Fig. S6 and S7). We focused this analysis on the WT, *cvnA8*, and *cvnF8* deletion strains. In both mutant strains, transcription at the 3′ end of the *aac(3)IV* cassette dropped to a very low level, irrespective of the genetic context. This steep decline in transcript abundance indicates that transcription directly downstream of the exchange site was not driven by promoters contained within the *aac(3)IV* cassette (Fig. S6). Furthermore, the pattern of independent transcriptional peaks within the *cvn8* and *lan* gene clusters downstream of the *aac(3)IV* cassette indicates that this transcription was not driven by promoters contained within the *aac(3)IV* cassette (Fig. S7). We attempted to complement the deletions, but the integration of different control plasmids into various integration sites, including φC31 and φBT1 altered pigmentation production in both the wild-type and mutant strains, rendering the complementation unreliable.

10.1128/mSystems.00281-21.3 FIG S3 Isolated patch phenotypes of S. coelicolor strains. Wild-type S. coelicolor and five mutant strains were spotted on ISP2 agar and grown for 4 days to observe the phenotypes of the strains as isolated patches. Download FIG S3, PDF file, 0.4 MB. Copyright © 2021 Bonet et al. 2021 Bonet et al. https://creativecommons.org/licenses/by/4.0/ This content is distributed under the terms of the Creative Commons Attribution 4.0 International license.

10.1128/mSystems.00281-21.4 FIG S4 Gene expression of the *cvn8* genes showing mutations are nonpolar. A heatmap with the log_2_ expression ratio of the genes in and surrounding *cvn8* in five S. coelicolor deletion strains compared to the wild type in both isolated and interaction growth conditions. Download FIG S4, PDF file, 0.3 MB. Copyright © 2021 Bonet et al. 2021 Bonet et al. https://creativecommons.org/licenses/by/4.0/ This content is distributed under the terms of the Creative Commons Attribution 4.0 International license.

10.1128/mSystems.00281-21.5 FIG S5 Gene expression of the *lan* genes in S. coelicolor interactions. A heatmap with the log_2_ expression ratio of the genes in a cryptic lanthipeptide biosynthetic gene cluster in S. coelicolor during interspecies interactions compared to S. coelicolor grown as an isolated patch. The gene numbers and their protein predictions are shown. Gray cells indicate genes that were not detected. These data are from the same data set as the data in Fig. 1. Download FIG S5, PDF file, 0.4 MB. Copyright © 2021 Bonet et al. 2021 Bonet et al. https://creativecommons.org/licenses/by/4.0/ This content is distributed under the terms of the Creative Commons Attribution 4.0 International license.

10.1128/mSystems.00281-21.6 FIG S6 mRNA read coverage of the *cvn8* and *lan* gene clusters in the wild type and *cvnA8* and *cvnF8* mutants during interspecies interactions. For this figure, the *y*-axis maxima are all set at 1,000 to enable direct comparison across strains. Mutation of either *cvnA8* or *cvnF8* resulted in much stronger transcription within both the *cvn8* and *lan* gene clusters (B and C) compared to transcription detected in the WT (A). Transcription at the 3′ end of the *aac(3)IV* cassette dropped below a coverage of 200 (denoted by black arrows in panels B and C), irrespective of the genetic context. This steep decline in transcript abundance indicates that transcription directly downstream of the exchange site was not driven by promoters contained within the *aac(3)IV* cassette. Note that nucleotide positions vary between panels A, B, and C due to *de novo* transcriptome assembly and differences resulting from allelic exchange with the *aac(3)IV* cassette. Download FIG S6, PDF file, 0.2 MB. Copyright © 2021 Bonet et al. 2021 Bonet et al. https://creativecommons.org/licenses/by/4.0/ This content is distributed under the terms of the Creative Commons Attribution 4.0 International license.

10.1128/mSystems.00281-21.7 FIG S7 mRNA read coverage of the *cvn8* and *lan* gene clusters in the wild type and *cvnA8* and *cvnF8* mutants during interspecies interactions. The same data are shown in Fig. S6; however, for this figure, the *y*-axis maxima are independent. Mutation of either *cvnA8* or *cvnF8* resulted in much stronger transcription within both the *cvn8* and *lan* gene clusters (B and C) compared to transcription detected in the WT (A). The pattern of independent transcriptional peaks downstream of the *aac(3)IV* cassette indicates that downstream transcription was not driven by promoters contained within the *aac(3)IV* cassette. Note that nucleotide positions vary between panels A, B, and C due to *de novo* transcriptome assembly and differences resulting from allelic exchange with the *aac(3)IV* cassette. Download FIG S7, PDF file, 0.2 MB. Copyright © 2021 Bonet et al. 2021 Bonet et al. https://creativecommons.org/licenses/by/4.0/ This content is distributed under the terms of the Creative Commons Attribution 4.0 International license.

### RNA isolation.

Biomass was collected after 4 days of growth at 30°C using a cell scraper. For the patches growing alone, the entire patch was collected, and one sample consists of three whole patches combined. For the patches in interactions, only half of the patch, the side closest to the initiator strain, was collected, and one sample consists of five half patches. Precaution was taken to not scrape up any of the interacting strain.

Immediately after collection, the biomass was frozen in liquid nitrogen and ground up using a mortar and pestle. Two hundred microliters of TRIzol was added to the ground cells. The Zymogen Direct-zol RNA miniprep kit (catalog no. R2051) was used to purify the RNA, with the optional DNase treatment step included. The purity of the RNA was verified using a NanoDrop One UV-Vis spectrophotometer to measure the 260/280 and 260/230 absorbance ratios. The RNA was quantified on a Qubit fluorometer using an Invitrogen Qubit RNA-HS assay kit (catalog no. Q32852).

### RNA sequencing and analysis.

RNA preparation was done by the University of California (UC) Berkeley Functional Genomics Laboratory. Briefly, rRNA was removed using the Illumina Ribo-Zero rRNA removal kit (bacteria), and the remaining RNA was prepared with a Wafergen PrepX RNA library prep kit for Illumina. Indexing PCR was performed using in-house dual indexed primers and 14 cycles. Samples were sequenced on an Illumina HiSeq 4000 using 100-bp paired-end reads or an Illumina NovaSeq 600 using 150-bp paired-end reads.

Reads were cleaned and trimmed using trimmomatic version 0.33.0, mapped to the genome using bowtie2 version 2.2.4.1, and counted using HTSeq version 0.11.3, and differential expression was tested using DESeq2 version 1.26.0 on R version 3.6.3. Each strain and environmental condition had three biological replicates.

For the coverage data analysis in Fig. S6 and S7, a reference genome was generated for each mutant by editing the S. coelicolor A3(2) reference genome to reflect the substitution of the deleted *cvn8* gene with *aac(3)IV*. Trimmed reads from Illumina sequencing were aligned to its respective reference genome using Geneious. Coverage data were exported, analyzed, and visualized using a custom Python script with Pandas and Matplotlib libraries.

### Pigmentation detection and quantification. (i) Pixel color distance calculation.

The pixel color distance calculation (PCDC) method is used to determine how similar a pixel color is to a given target color.

First, we converted all the pixels in an image from RGB (red, green, and blue) to CIELAB (International Commission on Illumination L*a*b*). We used the CIELAB color space because it represents our visual perception of colors more accurately than RGB. The CIELAB model expresses color as three values: L* for the lightness from black to white, a* from green to red, and b* from blue to yellow. Here we use the subscript λ to represent L*, α to represent a*, and β to represent b*.

Given two colors in the CIELAB color space, color *X* (*X*_λ_, *X*_α_, and *X*_β_) and a second color *Y* (*Y*_λ_, *Y*_α_, and *Y*_β_), we calculate the distance (*d*) between them using equation 1 and determine how similar two colors are with 0 being the exact same color, and 1 being most different.

(1) $$d=\sqrt{{\left(X_{\text{λ}}-Y_{\text{λ}}\right)}^{2}\;+\;{\left(X_{\text{α}}-\;Y_{\text{α}}\right)}^{2}\;+\;{\left(X_{\text{β}}-\;Y_{\text{β}}\right)}^{2}}$$

Since we would like the distances to be evenly distributed in the [0, 1] range, we normalized the distances for each pixel to the maximum distance *d*_max_ from the target color c. Given a target color *c*, when *c* is on the surface of a three-dimensional (3D) sphere with diameter 1, that color has a maximum distance (*d*_max_) of 1. If *c* is not on the surface of the sphere, but instead within the sphere, the maximum distance (*d*_max_) from any given color to *c* will be less than 1. Given this, we can normalize the value *d* from equation 1 using the following steps:

• Calculate *d*_max_ of target color *c*.
  o *d*_max_ is the distance from *c* to the center of the sphere (*L*, 50, *a*, 0, *b*, 0), plus the radius of the sphere (0.5).
• Calculate distance *d* between the color in a given pixel and the target color *c*.
• Normalize *d* to obtain distance to the pigmentation target color (*d_s_*) in the range [0, 1] using equation 2:
  (2) $$d_{s}=\;d/d_{\text{max}}$$

### (ii) Patch masking.

In images of interactions, *Amycolatopsis* sp. AA4 patches were carefully cropped out using Photoshop, ensuring only S. coelicolor patches were used in the pigmentation detection workflow.

Using the PCDC method described above, we first averaged 10 pixels from the background (agar) to determine that our target background color was 33393E. We then defined any pixel with a normalized distance of 0.15 or less from 33393E to be part of the background. With this, we convert the image to a binary mask, where every pixel is either black (part of the background) or white (part of the foreground).

To correct for any noisy white pixels that arise from small bubbles or imperfections in the agar, we counted the number of adjacent white pixels in the mask and if the count was less than 2% of the total pixels in the image, we considered them noise and turned them black. Similarly, there were also missing black pixels that were within the patch (not part of the background) that had a *d_s_* less than or equal to 0.15, which were removed from the mask. We used a similar counting method to identify these missing black pixels and if they were less than 20% of the total pixels in the image, we considered them part of the patch (not part of the background) and turned them white.

This method resulted in a black-and-white mask indicating where the S. coelicolor patch is (white pixels) and the background is (black pixels) in the image.

### (iii) Pigmentation calculation.

We used the PCDC method to calculate the distance of each pixel within the mask to the target pigmentation color 803D33 that corresponds to red pigmentation. The target pigmentation color was calculated by averaging 20 pixels from highly pigmented patches. We defined the pigmentation (*p*) using the distance to the pigmentation target color (*d_s_*) with equation 3:

(3) $$p=\text{ 1 }–\;d_{s}$$

### Baseline normalization.

We selected a region of interest (ROI) within the mask that is a rectangle centered vertically, with a height of 20% of the maximum patch height.

We wanted to differentiate red pigmentation from other components of the pixel color, such as brightness and glare, that otherwise would contribute to our pigmentation calculation. To do this, we first calculated the pigmentation in the ROI across 12 wild-type S. coelicolor isolated patches which are not pigmented. We grouped the columns of pixels in the ROI into breakpoints of 200 per image across the horizontal axis. We then averaged each of these 200 positional *P* values across all 12 images, resulting in an average pigmentation distribution of wild-type S. coelicolor isolated patches. Wild-type isolated patches were chosen because they have no visible pigmentation. This data set of spatial averages was named Pb, and Pb_min_ (0.342) was the minimum value in Pb distribution.

We then went back to our image of interest and adjusted the pigmentation value *p* in each pixel to give us evenly distributed values in the [0, 1] range, setting Pb_min_ to 0. Given a pigmentation value *p* we define the effective pixel pigmentation *P_e_* as:

$${\text{Pb}}_{\text{min}}=\;0.\text{342}$$

$$\mathrm{If}\;P<{\text{ Pb}}_{\text{min}},$$

$$P_{e}=0$$

$$\mathrm{If}\;P\geq {\text{ Pb}}_{\text{min}},$$

$$P_{e}=\;\left(P\;-{\text{ Pb}}_{\text{min}}\right)/\left(\text{1 }-{\text{ Pb}}_{\text{min}}\right)$$

Edges were often shadowed because of the three-dimensional aspect of the patch, and this created noise toward the edges of all the patches. In order to account for these edge effects, we used the Pb data set to apply a spatially specific pigmentation correction. Given the calculated *P_e_* of each pixel, we determined which breakpoint the given pixel falls in (*i*) and then subtracted the difference between the baseline pigmentation of that breakpoint Pb_i_ and Pb_min_ to give the final pigmentation value, *P_f_*, using equation 4:

(4) $$P_{f}=\;P_{e}-\;{\left({\text{Pb}}_{\text{i}}-{\text{ Pb}}_{\text{min}}\right)}_{\;\;}i\;=\;\left[\text{1},\text{2}00\right]$$

The *P_f_* values for the pixels in a given breakpoint were then averaged, and the standard deviation was determined.

### Data availability.

The software developed for pigmentation detection and quantification is available as a public repository on GitHub: https://github.com/TraxlerLab/ColonyPigmentationImageAnalysis.

The first RNA-seq data series detailed in this paper (Fig. 1) is available as a normalized data set and associated metafiles via Figshare: https://figshare.com/articles/dataset/Bonet_et_al_2021_RNAseq_Data_Series_1/14765640.

The second RNA-seq data set detailed in this paper (Fig. 4 and 6) can be found in the NCBI GEO database under SuperSeries accession no. GSE167966.
