# Supplementary material for: The cvn8 Conservon System Is a Global Regulator of Specialized Metabolism in Streptomyces coelicolor during Interspecies Interactions
Source: mSystems. 2021 Oct 12;6(5):e00281-21. doi: 10.1128/mSystems.00281-21 (PMC8510531; doi:10.1128/mSystems.00281-21)
Supplement: FIG S1 [file msystems.00281-21-sf001.pdf]

A

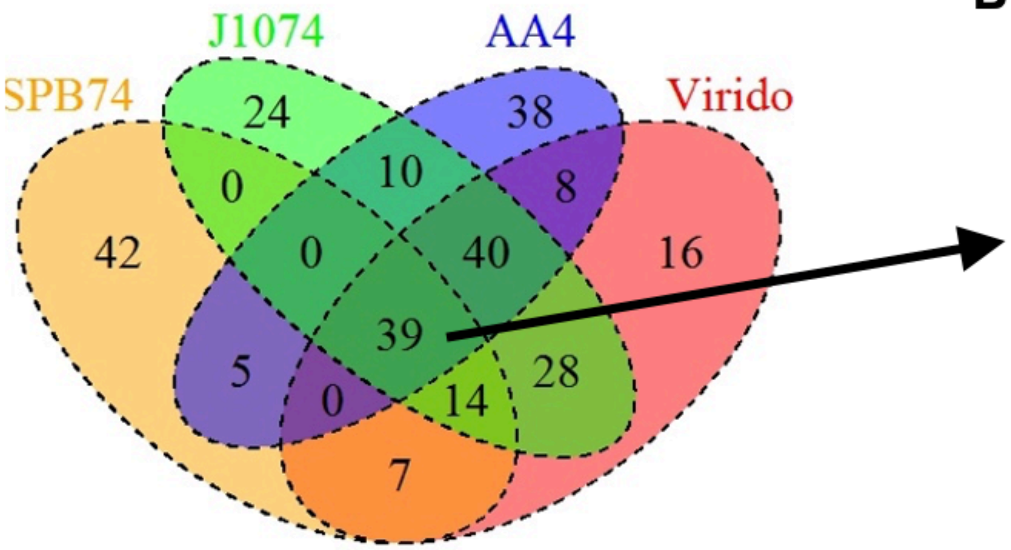

B

Localization or Function	Number of Genes
Secreted	8
Membrane associated	8
DNA binding	2
SM biosynthesis	10
Unknown	15

Figure S1. Four-way Venn diagram showing *S. coelicolor* genes significantly upregulated in the interactions (A), and the predicted localization or function of the genes significantly upregulated in all four interactions, determined by looking at their predicted domains or signal sequences (B). SM stands for specialized metabolite.
