# Supplementary material for: The cvn8 Conservon System Is a Global Regulator of Specialized Metabolism in Streptomyces coelicolor during Interspecies Interactions
Source: mSystems. 2021 Oct 12;6(5):e00281-21. doi: 10.1128/mSystems.00281-21 (PMC8510531; doi:10.1128/mSystems.00281-21)
Supplement: FIG S2 [file msystems.00281-21-sf002.pdf]

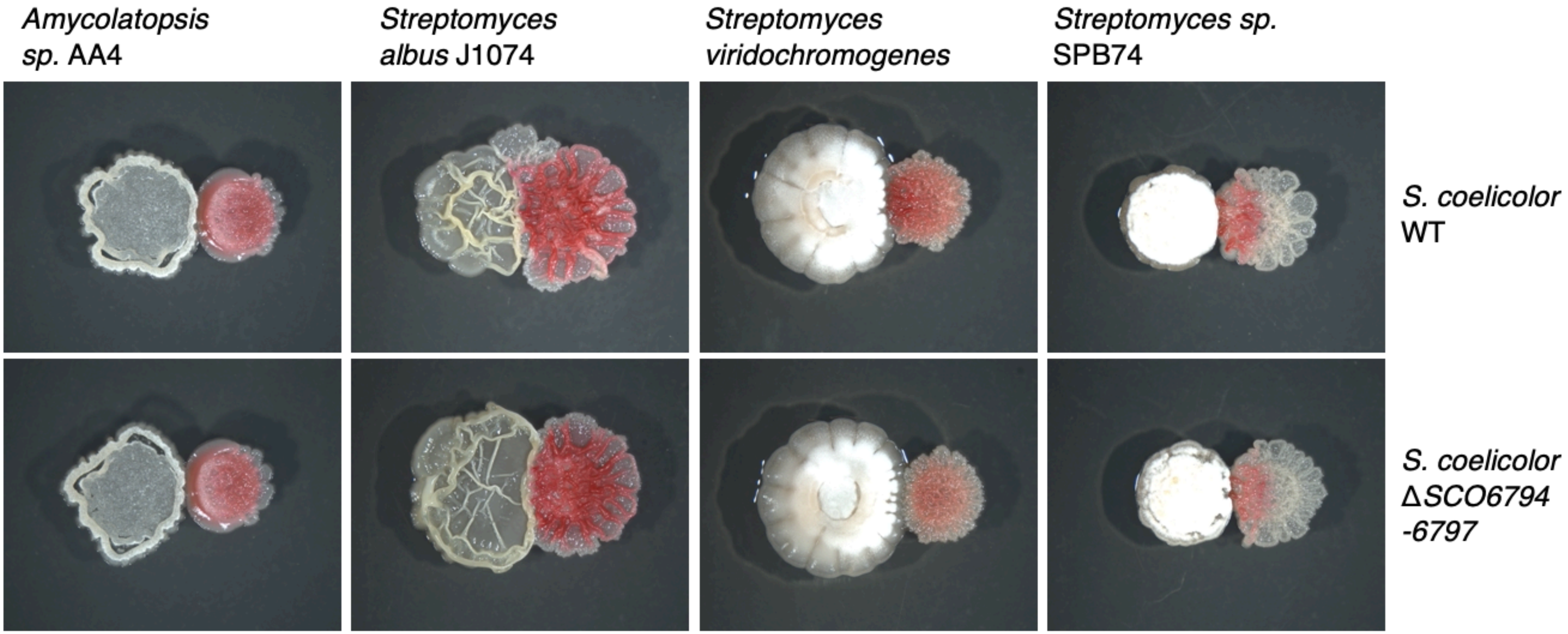

Figure S2. *Δcvn7* interaction phenotypes

Interspecies interactions of wild-type (WT) and Δ*cvn7* (Δ*SCO6794-6797*) *S. coelicolor* patches when grown in interspecies interactions with four interacting strains. Micrographs were taken after 5 days of growth for every interaction shown.
