# Supplementary material for: The cvn8 Conservon System Is a Global Regulator of Specialized Metabolism in Streptomyces coelicolor during Interspecies Interactions
Source: mSystems. 2021 Oct 12;6(5):e00281-21. doi: 10.1128/mSystems.00281-21 (PMC8510531; doi:10.1128/mSystems.00281-21)
Supplement: FIG S3 [file msystems.00281-21-sf003.pdf]

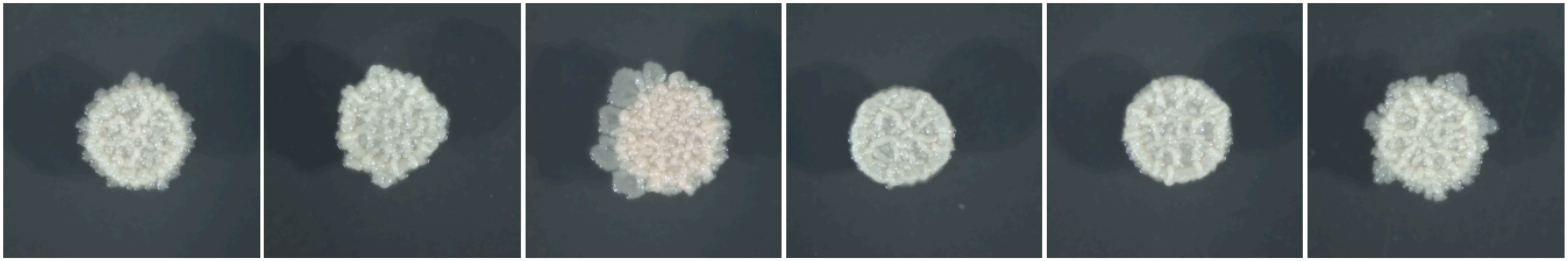

Figure S3. Isolated patch phenotypes of *S. coelicolor* strains.
Wild-type *S. coelicolor* and five mutants strains were spotted on ISP2 agar and grown for 4 days to observe the phenotypes of the strains as isolated patches.
