# Supplementary material for: The cvn8 Conservon System Is a Global Regulator of Specialized Metabolism in Streptomyces coelicolor during Interspecies Interactions
Source: mSystems. 2021 Oct 12;6(5):e00281-21. doi: 10.1128/mSystems.00281-21 (PMC8510531; doi:10.1128/mSystems.00281-21)
Supplement: FIG S4 [file msystems.00281-21-sf004.pdf]

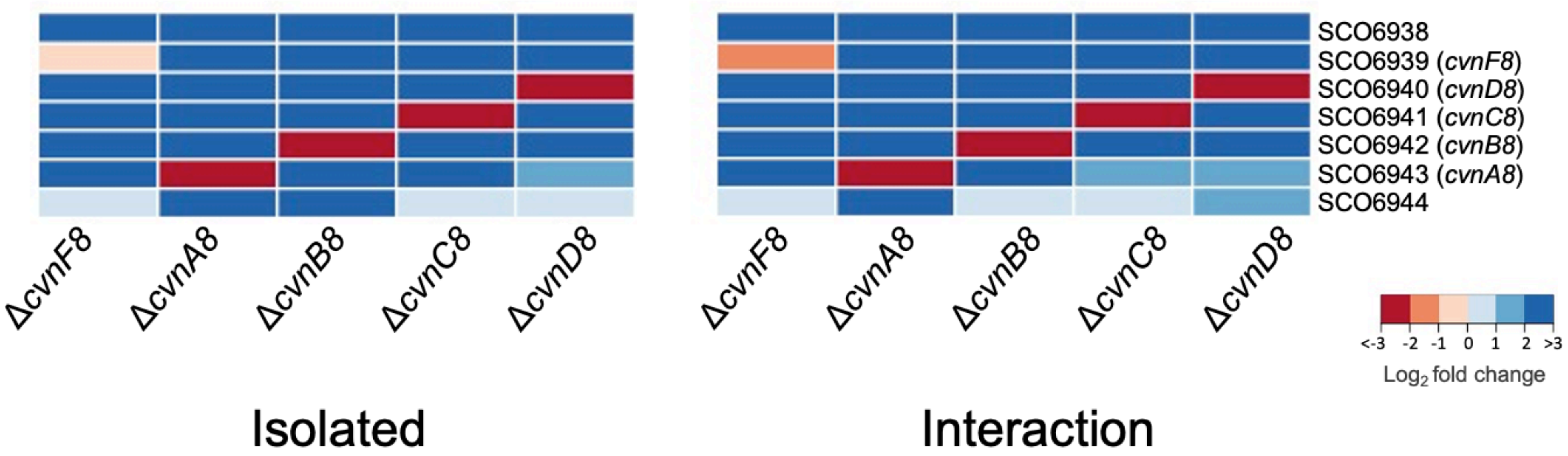

Figure S4. Gene expression of the *cvn8* genes showing mutations are non-polar

A heatmap with the $\log_2$ expression ratio of the genes in and surrounding *cvn8* in five *S. coelicolor* deletion strains compared to wild-type in both isolated and interaction growth conditions.
