# Supplementary material for: The cvn8 Conservon System Is a Global Regulator of Specialized Metabolism in Streptomyces coelicolor during Interspecies Interactions
Source: mSystems. 2021 Oct 12;6(5):e00281-21. doi: 10.1128/mSystems.00281-21 (PMC8510531; doi:10.1128/mSystems.00281-21)
Supplement: FIG S5 [file msystems.00281-21-sf005.pdf]

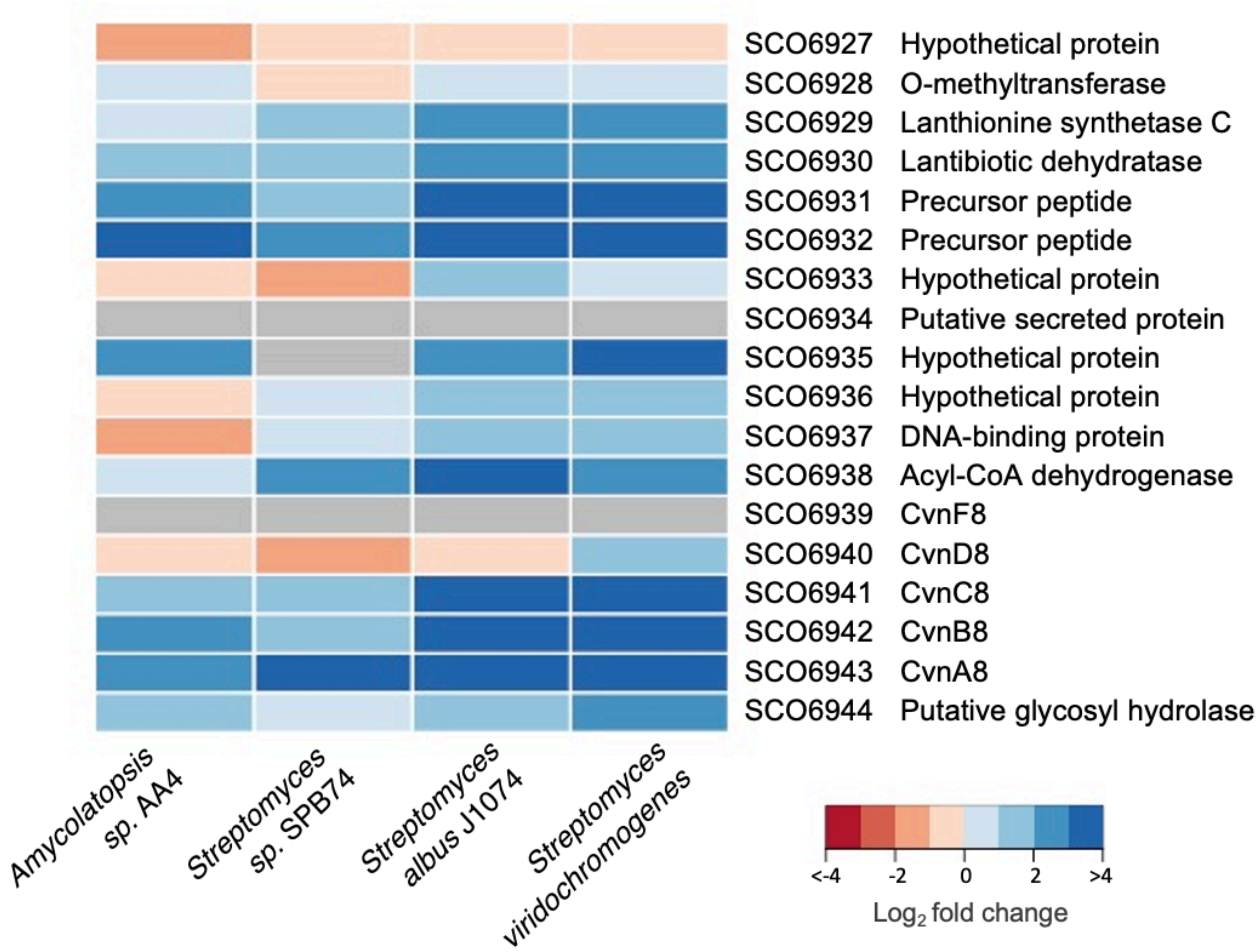

Figure S5. Gene expression of the *lan* genes in *S. coelicolor* interactions

A heatmap with the log_2 expression ratio of the genes in a cryptic lanthipeptide biosynthetic gene cluster in *S. coelicolor* during interspecies interactions compared to *S. coelicolor* grown as an isolated patch. The gene numbers and their protein predictions are shown. Gray cells indicate genes which were not detected. These data are from the same dataset as Figure 1.
