# Supplementary material for: The cvn8 Conservon System Is a Global Regulator of Specialized Metabolism in Streptomyces coelicolor during Interspecies Interactions
Source: mSystems. 2021 Oct 12;6(5):e00281-21. doi: 10.1128/mSystems.00281-21 (PMC8510531; doi:10.1128/mSystems.00281-21)
Supplement: FIG S6 [file msystems.00281-21-sf006.pdf]

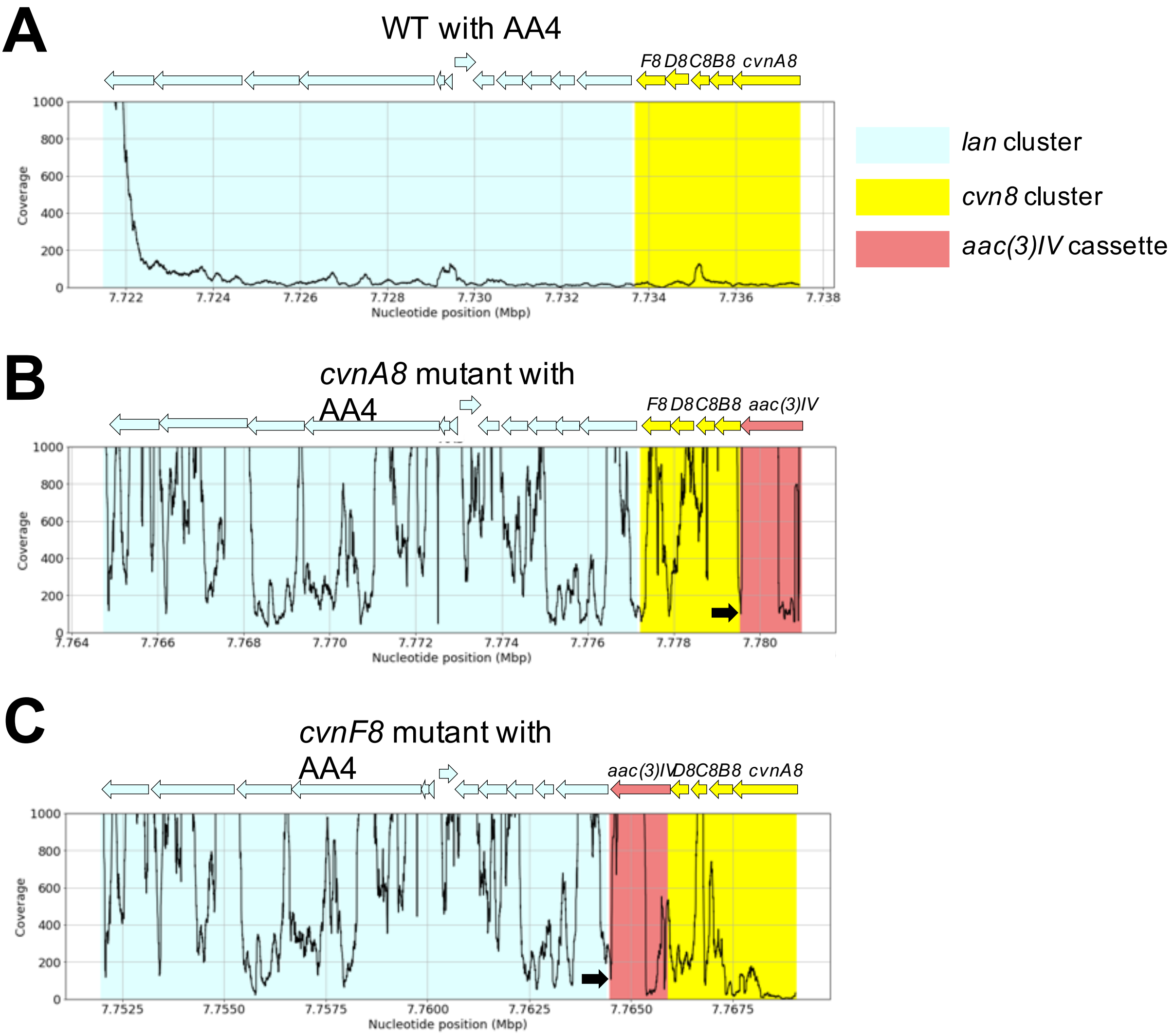

Figure S6. mRNA read coverage of the *cvn8* and *lan* gene clusters in wildtype, and *cvnA8* and *cvnF8* mutants during interspecies interactions. For this figure, the Y-axes maxima are all set at 1000 to enable direct comparison across strains. Mutation of either *cvnA8* or *cvnF8* resulted in much stronger transcription within both the *cvn8* and *lan* gene clusters (**B** and **C**), compared to transcription detected in the WT (**A**). Transcription at the 3' end of the *aac(3)IV* cassette dropped below a normalized coverage of 200 (denoted by black arrows in B and C), irrespective of the genetic context. This steep decline in transcript abundance indicates that transcription directly downstream of the exchange site was not driven by promoters contained within the *aac(3)IV* cassette. Note that nucleotide positions vary between A, B, and C, due to *de novo* transcriptome assembly and differences resulting from allelic exchange with the *aac(3)IV* cassette.
