# Supplementary material for: The cvn8 Conservon System Is a Global Regulator of Specialized Metabolism in Streptomyces coelicolor during Interspecies Interactions
Source: mSystems. 2021 Oct 12;6(5):e00281-21. doi: 10.1128/mSystems.00281-21 (PMC8510531; doi:10.1128/mSystems.00281-21)
Supplement: FIG S7 [file msystems.00281-21-sf007.pdf]

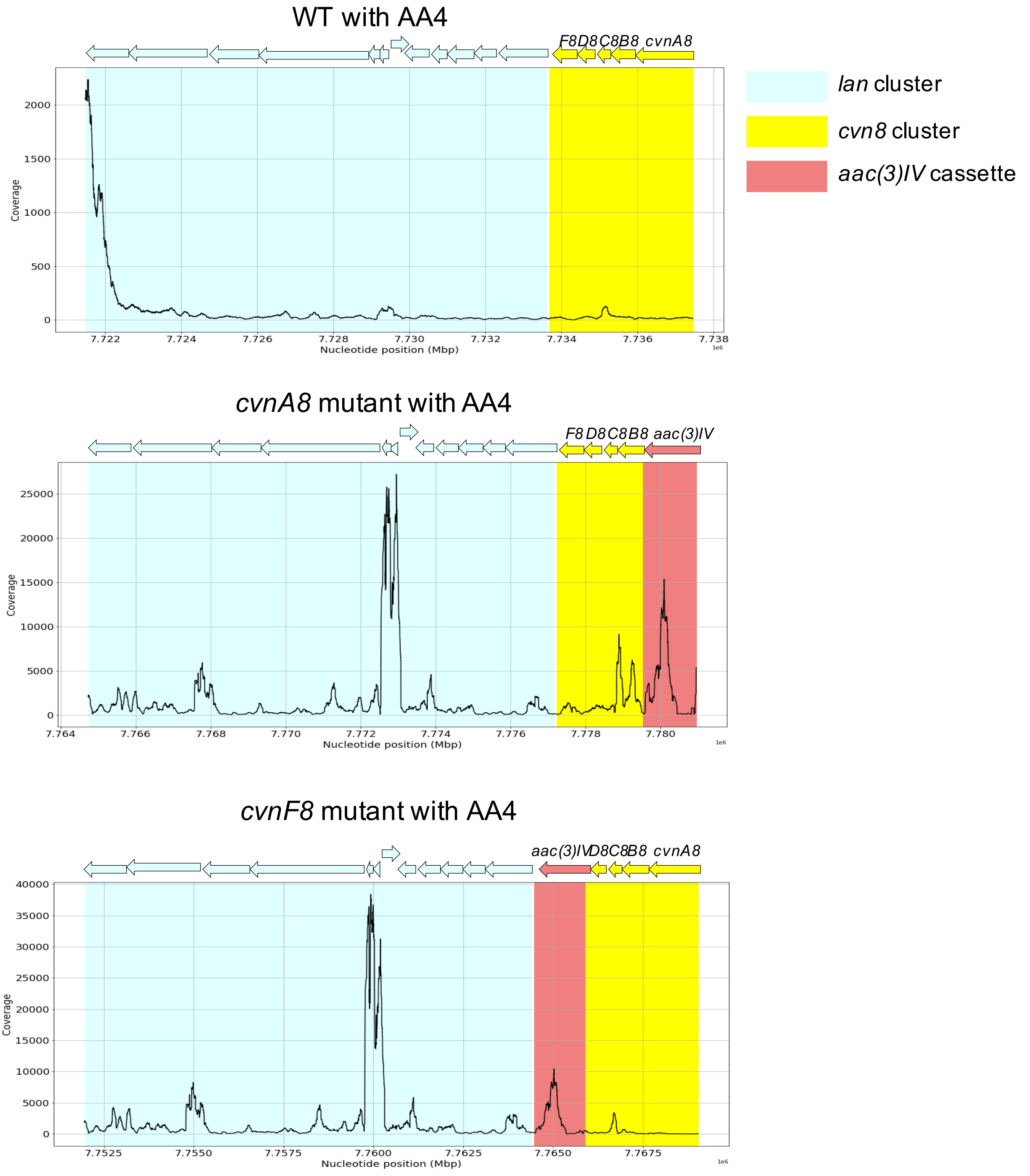

Figure S7. mRNA read coverage of the *cvn8* and *lan* gene clusters in wildtype, and *cvnA8* and *cvnF8* mutants during interspecies interactions. The same data are shown in Figure S6, however for this figure, the Y-axes maxima are independent. Mutation of either *cvnA8* or *cvnF8* resulted in much stronger transcription within both the *cvn8* and *lan* gene clusters (**B** and **C**) compared to transcription detected in the WT (**A**). The pattern of independent transcriptional peaks downstream of the *aac(3)IV* cassette indicates that downstream transcription was not driven by promoters contained within the *aac(3)IV* cassette. Note that nucleotide positions vary between A, B, and C, due to *de novo* transcriptome assembly and differences resulting from allelic exchange with the *aac(3)IV* cassette.
